# Supplementary material for: PVRIG Expression Is an Independent Prognostic Factor and a New Potential Target for Immunotherapy in Hepatocellular Carcinoma
Source: Cancers (Basel). 2023 Jan 10;15(2):447. doi: 10.3390/cancers15020447 (PMC9856571; doi:10.3390/cancers15020447)
Supplement: Supplementary file 1 [file cancers-15-00447-s001.zip › cancers-2094829-supplementary.pdf]

cancers

Supplementary Material

PVRIG Expression is an Independent Prognostic Factor and a New Target for Immunotherapy in Hepatocellular Carcinoma

David J Birnbaum, Maelle Picard, Quentin Da Costa, Thomas Delayre, Pascal Finetti, Olivier Cabaud, Emilie Agavnian, Bernadette de Rauglaudre, Emilie Denicolaï, François Bertucci, Emilie Mamessier

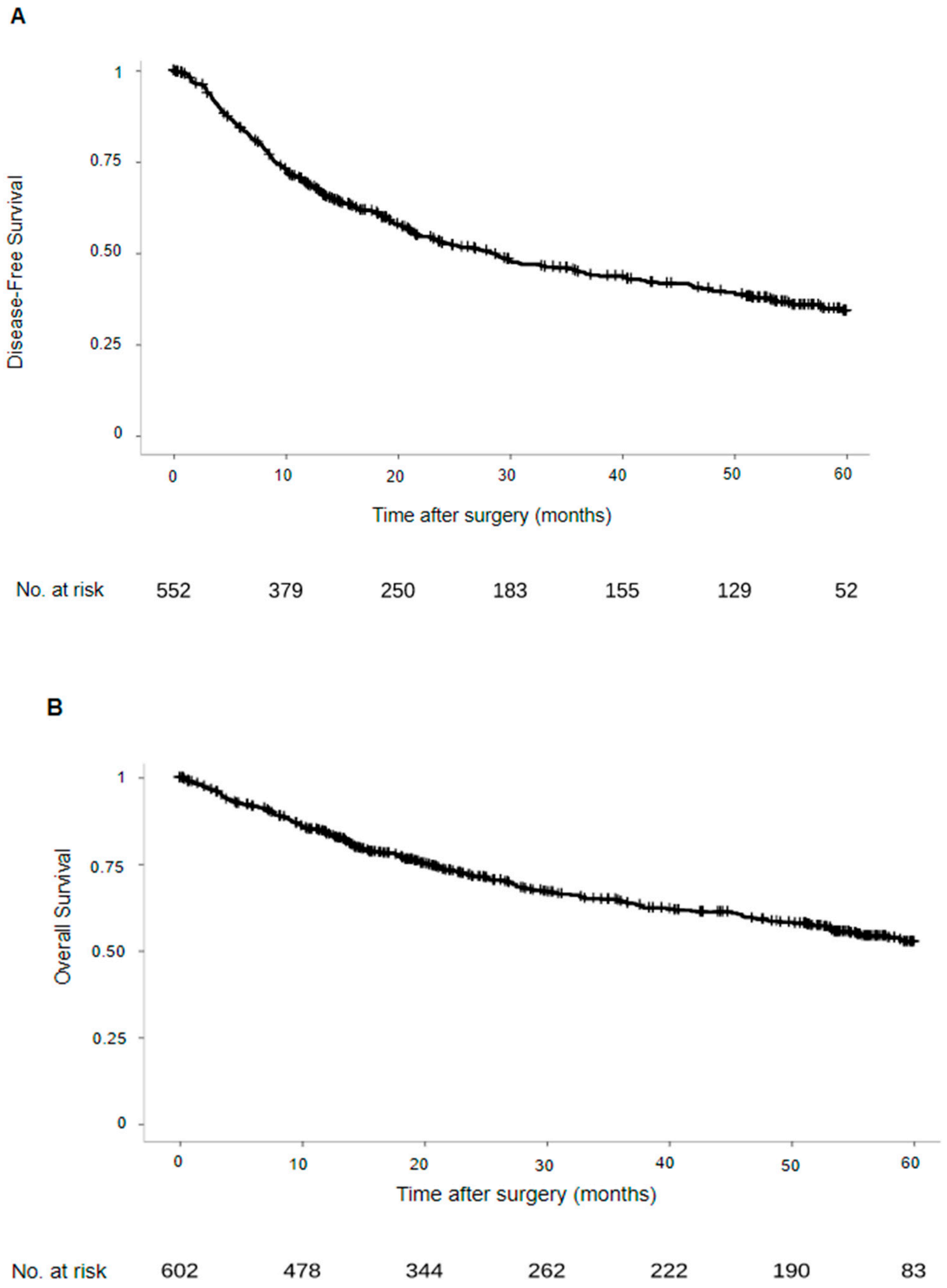

Figure S1. Survival in patient with HCC. (**A**) Kaplan-Meier DFS curve in the whole cohort of HCC patients (n=552). (**B**) Kaplan-Meier OS curve in the whole cohort of HCC patients (n=602). The p-value is for log rank test.

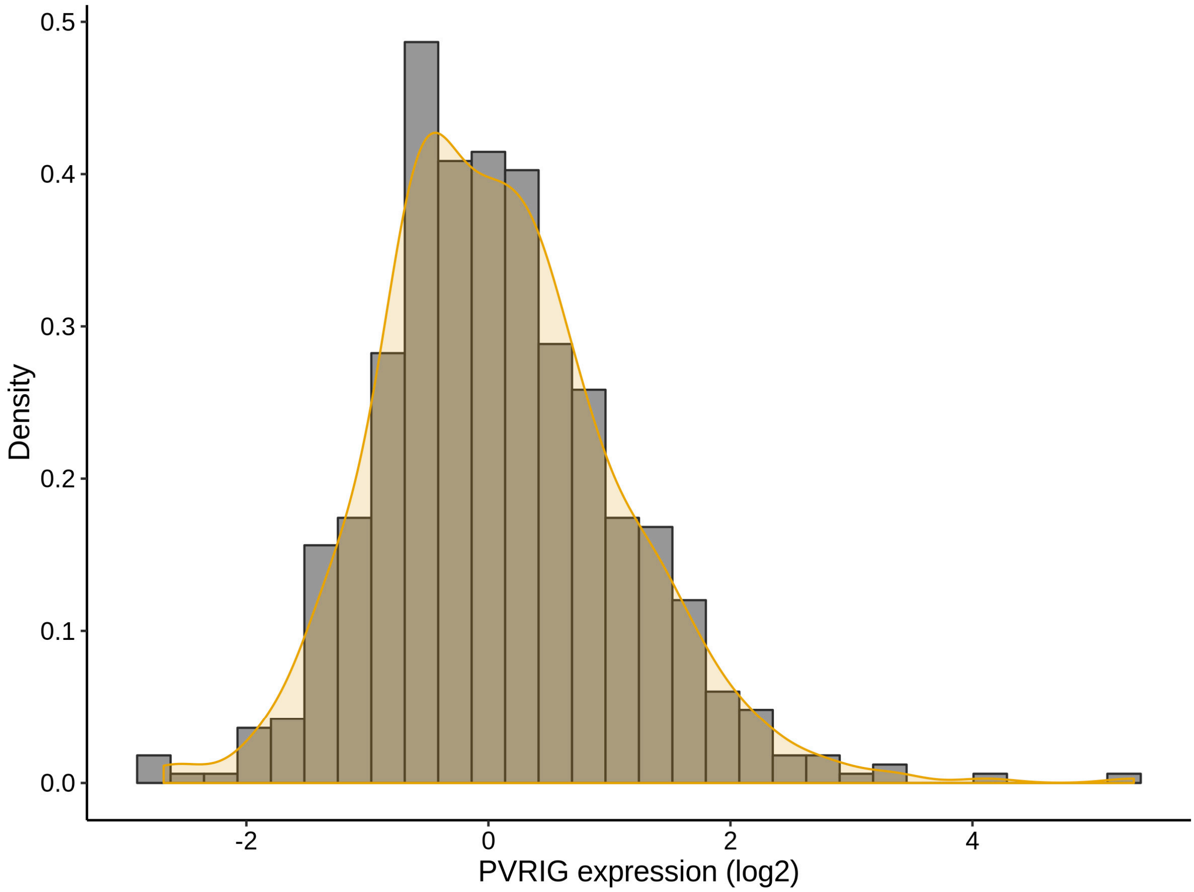

Figure S2: Distribution of *PVRIG* expression in HCC samples. Histogram of distribution of *PVRIG* mRNA expression levels (log2) across the 602 clinical samples. The orange line represents the density curve of distribution.

Table S1. **Data sets included in the study**

**Reference**	**Source of data**	**Samples (N)**	**HCC samples (n)**	**healthy liver samples (n)**	**Technological platform**
The Cancer Genome Atlas Research Network. *Cell* 2017	https://www.cbioportal.org/	360	360	0	Illumina HiSeq RNA-seq
Roessler et al., *Cancer Res* 2010	GEO: GSE14520	481	242	239	Affymetrix Human Genome U133A 2.0 Array; Affymetrix HT Human Genome U133A Array
Villa et al., *Gut* 206	GEO: GSE54236	161	81	80	Agilent-014850 Whole Human Genome Microarray 4x44K G4112F
**Total**		**1002**	**683**	**319**	

Table S2. Association between expression of members of TIGIT/DNAM-1 axis and PD-1/PD-L1 axis related RNA expression and survival in HCC and survival in HCC

Gene symbol	Overall Survival		Disease-Free Survival	
	Univariate	Multivariate	Univariate	Multivariate
DNAM-1	**2.85×10^{-2}**	0.52	**1.03×10^{-3}**	0.14
PD-L1	0.51	-	0.67	-
TACTILE	**1.56×10^{-2}**	0.57	**7.17×10^{-4}**	0.77
CTLA-4	**4.87×10^{-2}**	0.79	**5.08×10^{-3}**	0.49
KIR2DL5A	0.18	-	0.19	-
NECTIN-1	**1.84×10^{-2}**	**5.48×10^{-3}**	6.38×10^{-2}	-
NECTIN-2	0.98	-	0.41	-
NECTIN-3	0.40	-	**2.16×10^{-2}**	0.61
PD-1	**3.48×10^{-2}**	0.20	**1.21×10^{-3}**	0.36
PVR	7.28×10^{-2}	-	0.66	-
PVRIG	**3.16×10^{-6}**	**4.57×10^{-4}**	**1.43×10^{-4}**	0.37
TIGIT	0.22	-	**2.98×10^{-2}**	0.85

Table S3. List of the 181 genes differentially expressed between *PVRIG* High and Low groups in the learning set (GSE14520, n=242)

Entrez ID	Symbol	Full name	Genomic loc	NCBI link	Fold change	p-value	Adjusted p-value	Upregulated (Red/Green)
79037	PVRIG	PVR related immunoglobulin domain containing	7q22.1	https://www.ncbi.nlm.nih.gov/gene/?term=79037+AND+homo+sapiens	1.52	**1.0E-38**	1.2E-34	PVRIG_High
914	CD2	CD2 molecule	1p13.1	https://www.ncbi.nlm.nih.gov/gene/?term=914+AND+homo+sapiens	2.48	**1.3E-19**	7.1E-16	PVRIG_High
1043	CD52	CD52 molecule	1p36.11	https://www.ncbi.nlm.nih.gov/gene/?term=1043+AND+homo+sapiens	2.45	**1.3E-18**	3.9E-15	PVRIG_High
962	CD48	CD48 molecule	1q23.3	https://www.ncbi.nlm.nih.gov/gene/?term=962+AND+homo+sapiens	2.14	**1.7E-18**	3.9E-15	PVRIG_High
5880	RAC2	Rac family small GTPase 2	22q13.1	https://www.ncbi.nlm.nih.gov/gene/?term=5880+AND+homo+sapiens	2.21	**6.9E-18**	1.3E-14	PVRIG_High
80342	TRAF3IP3	TRAF3 interacting protein 3	1q32.2	https://www.ncbi.nlm.nih.gov/gene/?term=80342+AND+homo+sapiens	1.67	**2.5E-17**	4.1E-14	PVRIG_High
11151	CORO1A	coronin 1A	16p11.2	https://www.ncbi.nlm.nih.gov/gene/?term=11151+AND+homo+sapiens	2.36	**3.8E-17**	4.9E-14	PVRIG_High
6352	CCL5	C-C motif chemokine ligand 5	17q12	https://www.ncbi.nlm.nih.gov/gene/?term=6352+AND+homo+sapiens	2.95	**3.9E-17**	4.9E-14	PVRIG_High
10125	RASGRP1	RAS guanyl releasing protein 1	15q14	https://www.ncbi.nlm.nih.gov/gene/?term=10125+AND+homo+sapiens	1.94	**8.9E-17**	8.4E-14	PVRIG_High
10659	CELF2	CUGBP Elav-like family member 2	10p14	https://www.ncbi.nlm.nih.gov/gene/?term=10659+AND+homo+sapiens	2.25	**2.2E-16**	1.9E-13	PVRIG_High
6404	SELPLG	selectin P ligand	12q24.11	https://www.ncbi.nlm.nih.gov/gene/?term=6404+AND+homo+sapiens	1.64	**3.3E-16**	2.5E-13	PVRIG_High
3001	GZMA	granzyme A	5q11.2	https://www.ncbi.nlm.nih.gov/gene/?term=3001+AND+homo+sapiens	2.00	**4.5E-16**	3.2E-13	PVRIG_High
3003	GZMK	granzyme K	5q11.2	https://www.ncbi.nlm.nih.gov/gene/?term=3003+AND+homo+sapiens	1.85	**5.5E-16**	3.6E-13	PVRIG_High

5790	PTPRC AP	protein tyrosine phosphatase, receptor type C associated protein	11q13.2	https://www.ncbi.nlm.nih.gov/gene/?term=5790+AND+homo+sapiens	1.56	**6.0E-16**	3.6E-13	PVRIG_High
3059	HCLS1	hematopoietic cell-specific Lyn substrate 1	3q13.33	https://www.ncbi.nlm.nih.gov/gene/?term=3059+AND+homo+sapiens	1.94	**7.7E-15**	3.8E-12	PVRIG_High
925	CD8A	CD8a molecule	2p11.2	https://www.ncbi.nlm.nih.gov/gene/?term=925+AND+homo+sapiens	2.60	**8.6E-15**	4.0E-12	PVRIG_High
9806	SPOCK2	SPARC (osteonectin), cwcv and kazal like domains proteoglycan 2	10q22.1	https://www.ncbi.nlm.nih.gov/gene/?term=9806+AND+homo+sapiens	1.64	**9.2E-15**	4.0E-12	PVRIG_High
8530	CST7	cystatin F	20p11.21	https://www.ncbi.nlm.nih.gov/gene/?term=8530+AND+homo+sapiens	1.61	**9.3E-15**	4.0E-12	PVRIG_High
2533	FYB1	FYN binding protein 1	5p13.1	https://www.ncbi.nlm.nih.gov/gene/?term=2533+AND+homo+sapiens	1.65	**1.2E-14**	5.1E-12	PVRIG_High
3937	LCP2	lymphocyte cytosolic protein 2	5q35.1	https://www.ncbi.nlm.nih.gov/gene/?term=3937+AND+homo+sapiens	1.67	**1.4E-14**	5.7E-12	PVRIG_High
3561	IL2RG	interleukin 2 receptor subunit gamma	Xq13.1	https://www.ncbi.nlm.nih.gov/gene/?term=3561+AND+homo+sapiens	2.17	**2.0E-14**	7.7E-12	PVRIG_High
5579	PRKCB	protein kinase C beta	16p12.2-p12.1	https://www.ncbi.nlm.nih.gov/gene/?term=5579+AND+homo+sapiens	1.55	**2.7E-14**	1.0E-11	PVRIG_High
22914	KLRK1	killer cell lectin like receptor K1	12p13.2	https://www.ncbi.nlm.nih.gov/gene/?term=22914+AND+homo+sapiens	1.80	**3.6E-14**	1.3E-11	PVRIG_High
445347	TARP	TCR gamma alternate reading frame protein	7p14.1	https://www.ncbi.nlm.nih.gov/gene/?term=445347+AND+homo+sapiens	1.64	**4.1E-14**	1.4E-11	PVRIG_High
5788	PTPRC	protein tyrosine phosphatase, receptor type C	1q31.3-q32.1	https://www.ncbi.nlm.nih.gov/gene/?term=5788+AND+homo+sapiens	2.50	**4.2E-14**	1.4E-11	PVRIG_High

5293	PIK3CD	phosphatidylinositol-4,5-bisphosphate 3-kinase catalytic subunit delta	1p36.22	https://www.ncbi.nlm.nih.gov/gene/?term=5293+AND+homo+sapiens	1.78	**5.2E-14**	1.7E-11	PVRIG_High
4046	LSP1	lymphocyte-specific protein 1	11p15.5	https://www.ncbi.nlm.nih.gov/gene/?term=4046+AND+homo+sapiens	1.51	**7.4E-14**	2.3E-11	PVRIG_High
3560	IL2RB	interleukin 2 receptor subunit beta	22q12.3	https://www.ncbi.nlm.nih.gov/gene/?term=3560+AND+homo+sapiens	1.61	**7.8E-14**	2.3E-11	PVRIG_High
3932	LCK	LCK proto-oncogene, Src family tyrosine kinase	1p35.2	https://www.ncbi.nlm.nih.gov/gene/?term=3932+AND+homo+sapiens	1.56	**7.9E-14**	2.3E-11	PVRIG_High
9595	CYTIP	cytohesin 1 interacting protein	2q24.1	https://www.ncbi.nlm.nih.gov/gene/?term=9595+AND+homo+sapiens	1.87	**9.7E-14**	2.7E-11	PVRIG_High
969	CD69	CD69 molecule	12p13.31	https://www.ncbi.nlm.nih.gov/gene/?term=969+AND+homo+sapiens	1.67	**9.9E-14**	2.7E-11	PVRIG_High
963	CD53	CD53 molecule	1p13.3	https://www.ncbi.nlm.nih.gov/gene/?term=963+AND+homo+sapiens	2.22	**1.1E-13**	3.0E-11	PVRIG_High
5450	POU2AF1	POU class 2 associating factor 1	11q23.1	https://www.ncbi.nlm.nih.gov/gene/?term=5450+AND+homo+sapiens	3.28	**1.6E-13**	3.9E-11	PVRIG_High
3689	ITGB2	integrin subunit beta 2	21q22.3	https://www.ncbi.nlm.nih.gov/gene/?term=3689+AND+homo+sapiens	2.07	**2.4E-13**	6.0E-11	PVRIG_High
4050	LTB	lymphotoxin beta	6p21.33	https://www.ncbi.nlm.nih.gov/gene/?term=4050+AND+homo+sapiens	1.68	**2.7E-13**	6.6E-11	PVRIG_High
55303	GIMAP4	GTPase, IMAP family member 4	7q36.1	https://www.ncbi.nlm.nih.gov/gene/?term=55303+AND+homo+sapiens	1.61	**7.2E-13**	1.6E-10	PVRIG_High
6351	CCL4	C-C motif chemokine ligand 4	17q12	https://www.ncbi.nlm.nih.gov/gene/?term=6351+AND+homo+sapiens	2.00	**9.2E-13**	2.0E-10	PVRIG_High
4818	NKG7	natural killer cell granule protein 7	19q13.41	https://www.ncbi.nlm.nih.gov/gene/?term=4818+AND+homo+sapiens	1.86	**9.3E-13**	2.0E-10	PVRIG_High
6503	SLA	Src like adaptor	8q24.22	https://www.ncbi.nlm.nih.gov/gene/?term=6503+AND+homo+sapiens	1.72	**1.2E-12**	2.6E-10	PVRIG_High

54440	SASH3	SAM and SH3 domain containing 3	Xq26.1	https://www.ncbi.nlm.nih.gov/gene/?term=54440+AND+homo+sapiens	1.58	**1.8E-12**	3.8E-10	**PVRIG_High**
3512	JCHAIN	joining chain of multimeric IgA and IgM	4q13.3	https://www.ncbi.nlm.nih.gov/gene/?term=3512+AND+homo+sapiens	4.06	**2.3E-12**	4.6E-10	**PVRIG_High**
199	AIF1	allograft inflammatory factor 1	6p21.33	https://www.ncbi.nlm.nih.gov/gene/?term=199+AND+homo+sapiens	1.64	**3.9E-12**	7.7E-10	**PVRIG_High**
4283	CXCL9	C-X-C motif chemokine ligand 9	4q21.1	https://www.ncbi.nlm.nih.gov/gene/?term=4283+AND+homo+sapiens	4.04	**8.7E-12**	1.6E-09	**PVRIG_High**
9535	GMFG	glia maturation factor gamma	19q13.2	https://www.ncbi.nlm.nih.gov/gene/?term=9535+AND+homo+sapiens	1.69	**9.2E-12**	1.7E-09	**PVRIG_High**
6402	SELL	selectin L	1q24.2	https://www.ncbi.nlm.nih.gov/gene/?term=6402+AND+homo+sapiens	1.60	**1.0E-11**	1.8E-09	**PVRIG_High**
2123	EVI2A	ecotropic viral integration site 2A	17q11.2	https://www.ncbi.nlm.nih.gov/gene/?term=2123+AND+homo+sapiens	1.66	**1.2E-11**	2.0E-09	**PVRIG_High**
7351	UCP2	uncoupling protein 2	11q13.4	https://www.ncbi.nlm.nih.gov/gene/?term=7351+AND+homo+sapiens	2.06	**1.4E-11**	2.4E-09	**PVRIG_High**
712	C1QA	complement C1q A chain	1p36.12	https://www.ncbi.nlm.nih.gov/gene/?term=712+AND+homo+sapiens	2.18	**1.5E-11**	2.6E-09	**PVRIG_High**
113	ADCY7	adenylate cyclase 7	16q12.1	https://www.ncbi.nlm.nih.gov/gene/?term=113+AND+homo+sapiens	1.51	**1.7E-11**	2.9E-09	**PVRIG_High**
5341	PLEK	pleckstrin	2p14	https://www.ncbi.nlm.nih.gov/gene/?term=5341+AND+homo+sapiens	2.11	**1.8E-11**	3.0E-09	**PVRIG_High**
2124	EVI2B	ecotropic viral integration site 2B	17q11.2	https://www.ncbi.nlm.nih.gov/gene/?term=2124+AND+homo+sapiens	2.03	**2.6E-11**	4.0E-09	**PVRIG_High**
7456	WIPF1	WAS/WASL interacting protein family member 1	2q31.1	https://www.ncbi.nlm.nih.gov/gene/?term=7456+AND+homo+sapiens	1.83	**3.9E-11**	5.9E-09	**PVRIG_High**
4065	LY75	lymphocyte antigen 75	2q24.2	https://www.ncbi.nlm.nih.gov/gene/?term=4065+AND+homo+sapiens	1.97	**4.3E-11**	6.4E-09	**PVRIG_High**

6772	STAT1	signal transducer and activator of transcription 1	2q32.2	https://www.ncbi.nlm.nih.gov/gene/?term=6772+AND+homo+sapiens	1.94	**4.6E-11**	6.6E-09	PVRIG_High
6614	SIG-LEC1	sialic acid binding Ig like lectin 1	20p13	https://www.ncbi.nlm.nih.gov/gene/?term=6614+AND+homo+sapiens	1.50	**5.3E-11**	7.5E-09	PVRIG_High
5551	PRF1	perforin 1	10q22.1	https://www.ncbi.nlm.nih.gov/gene/?term=5551+AND+homo+sapiens	1.71	**7.2E-11**	9.6E-09	PVRIG_High
3587	IL10RA	interleukin 10 receptor subunit alpha	11q23.3	https://www.ncbi.nlm.nih.gov/gene/?term=3587+AND+homo+sapiens	1.82	**9.4E-11**	1.2E-08	PVRIG_High
397	ARHG-DIB	Rho GDP dissociation inhibitor beta	12p12.3	https://www.ncbi.nlm.nih.gov/gene/?term=397+AND+homo+sapiens	1.70	**1.1E-10**	1.4E-08	PVRIG_High
51237	MZB1	marginal zone B and B1 cell specific protein	5q31.2	https://www.ncbi.nlm.nih.gov/gene/?term=51237+AND+homo+sapiens	1.70	**1.1E-10**	1.4E-08	PVRIG_High
56833	SLAMF 8	SLAM family member 8	1q23.2	https://www.ncbi.nlm.nih.gov/gene/?term=56833+AND+homo+sapiens	1.75	**1.5E-10**	1.8E-08	PVRIG_High
64092	SAMSN 1	SAM domain, SH3 domain and nuclear localization signals 1	21q11.2	https://www.ncbi.nlm.nih.gov/gene/?term=64092+AND+homo+sapiens	1.97	**1.5E-10**	1.9E-08	PVRIG_High
713	C1QB	complement C1q B chain	1p36.12	https://www.ncbi.nlm.nih.gov/gene/?term=713+AND+homo+sapiens	2.17	**1.6E-10**	1.9E-08	PVRIG_High
7805	LAPTM 5	lysosomal protein transmembrane 5	1p35.2	https://www.ncbi.nlm.nih.gov/gene/?term=7805+AND+homo+sapiens	1.91	**1.6E-10**	1.9E-08	PVRIG_High
9672	SDC3	syndecan 3	1p35.2	https://www.ncbi.nlm.nih.gov/gene/?term=9672+AND+homo+sapiens	1.55	**1.7E-10**	2.0E-08	PVRIG_High
915	CD3D	CD3d molecule	11q23.3	https://www.ncbi.nlm.nih.gov/gene/?term=915+AND+homo+sapiens	1.67	**2.4E-10**	2.8E-08	PVRIG_High
23406	COTL1	coactosin like F-actin binding protein 1	16q24.1	https://www.ncbi.nlm.nih.gov/gene/?term=23406+AND+homo+sapiens	1.69	**3.6E-10**	4.1E-08	PVRIG_High

55340	GIMAP 5	GTPase, IMAP family member 5	7q36.1	https://www.ncbi.nlm.nih.gov/gene/?term=55340+AND+homo+sapiens	1.64	**7.5E-10**	8.0E-08	PVRIG_High
60489	APO-BEC3G	apolipoprotein B mRNA editing enzyme catalytic subunit 3G	22q13.1	https://www.ncbi.nlm.nih.gov/gene/?term=60489+AND+homo+sapiens	1.87	**9.1E-10**	9.5E-08	PVRIG_High
7453	WARS	tryptophanyl-tRNA synthetase	14q32.2	https://www.ncbi.nlm.nih.gov/gene/?term=7453+AND+homo+sapiens	1.99	**1.2E-09**	1.2E-07	PVRIG_High
10875	FGL2	fibrinogen like 2	7q11.23	https://www.ncbi.nlm.nih.gov/gene/?term=10875+AND+homo+sapiens	1.58	**1.3E-09**	1.2E-07	PVRIG_High
3115	HLA-DPB1	major histocompatibility complex, class II, DP beta 1	6p21.32	https://www.ncbi.nlm.nih.gov/gene/?term=3115+AND+homo+sapiens	1.77	**1.8E-09**	1.7E-07	PVRIG_High
7305	TY-ROBP	TYRO protein tyrosine kinase binding protein	19q13.12	https://www.ncbi.nlm.nih.gov/gene/?term=7305+AND+homo+sapiens	1.82	**2.2E-09**	2.1E-07	PVRIG_High
1230	CCR1	C-C motif chemokine receptor 1	3p21.31	https://www.ncbi.nlm.nih.gov/gene/?term=1230+AND+homo+sapiens	1.67	**4.9E-09**	4.3E-07	PVRIG_High
3002	GZMB	granzyme B	14q12	https://www.ncbi.nlm.nih.gov/gene/?term=3002+AND+homo+sapiens	1.66	**5.5E-09**	4.8E-07	PVRIG_High
51816	ADA2	adenosine deaminase 2	22q11.1	https://www.ncbi.nlm.nih.gov/gene/?term=51816+AND+homo+sapiens	1.80	**6.8E-09**	5.9E-07	PVRIG_High
6001	RGS10	regulator of G protein signaling 10	10q26.11	https://www.ncbi.nlm.nih.gov/gene/?term=6001+AND+homo+sapiens	1.72	**7.6E-09**	6.5E-07	PVRIG_High
9447	AIM2	absent in melanoma 2	1q23.1-q23.2	https://www.ncbi.nlm.nih.gov/gene/?term=9447+AND+homo+sapiens	1.54	**8.1E-09**	6.9E-07	PVRIG_High
6890	TAP1	transporter 1, ATP binding cassette subfamily B member	6p21.32	https://www.ncbi.nlm.nih.gov/gene/?term=6890+AND+homo+sapiens	1.76	**1.1E-08**	8.7E-07	PVRIG_High
55843	ARHGAP15	Rho GTPase activating protein 15	2q22.2-q22.3	https://www.ncbi.nlm.nih.gov/gene/?term=55843+AND+homo+sapiens	1.51	**1.1E-08**	9.0E-07	PVRIG_High

100132417	FCGR1CP	Fc fragment of IgG receptor Ic, pseudogene	1q21.1	https://www.ncbi.nlm.nih.gov/gene/?term=100132417+AND+homo+sapiens	1.67	**1.2E-08**	9.2E-07	PVRIG_High
64581	CLEC7A	C-type lectin domain containing 7A	12p13.2	https://www.ncbi.nlm.nih.gov/gene/?term=64581+AND+homo+sapiens	1.53	**1.2E-08**	9.6E-07	PVRIG_High
3659	IRF1	interferon regulatory factor 1	5q31.1	https://www.ncbi.nlm.nih.gov/gene/?term=3659+AND+homo+sapiens	1.59	**1.3E-08**	1.0E-06	PVRIG_High
3957	LGALS2	galectin 2	22q13.1	https://www.ncbi.nlm.nih.gov/gene/?term=3957+AND+homo+sapiens	1.51	**1.5E-08**	1.2E-06	PVRIG_High
3575	IL7R	interleukin 7 receptor	5p13.2	https://www.ncbi.nlm.nih.gov/gene/?term=3575+AND+homo+sapiens	1.78	**2.0E-08**	1.5E-06	PVRIG_High
27299	ADAMDEC1	ADAM like decysin 1	8p21.2	https://www.ncbi.nlm.nih.gov/gene/?term=27299+AND+homo+sapiens	2.00	**2.5E-08**	1.8E-06	PVRIG_High
4318	MMP9	matrix metallopeptidase 9	20q13.12	https://www.ncbi.nlm.nih.gov/gene/?term=4318+AND+homo+sapiens	2.13	**2.7E-08**	1.9E-06	PVRIG_High
6363	CCL19	C-C motif chemokine ligand 19	9p13.3	https://www.ncbi.nlm.nih.gov/gene/?term=6363+AND+homo+sapiens	3.83	**4.4E-08**	3.0E-06	PVRIG_High
6373	CXCL11	C-X-C motif chemokine ligand 11	4q21.1	https://www.ncbi.nlm.nih.gov/gene/?term=6373+AND+homo+sapiens	2.99	**5.2E-08**	3.5E-06	PVRIG_High
1536	CYBB	cytochrome b-245 beta chain	Xp21.1-p11.4	https://www.ncbi.nlm.nih.gov/gene/?term=1536+AND+homo+sapiens	1.68	**5.3E-08**	3.6E-06	PVRIG_High
51316	PLAC8	placenta specific 8	4q21.22	https://www.ncbi.nlm.nih.gov/gene/?term=51316+AND+homo+sapiens	1.97	**5.5E-08**	3.7E-06	PVRIG_High
25816	TNFAIP8	TNF alpha induced protein 8	5q23.1	https://www.ncbi.nlm.nih.gov/gene/?term=25816+AND+homo+sapiens	1.67	**6.4E-08**	4.2E-06	PVRIG_High
1439	CSF2RB	colony stimulating factor 2 receptor beta common subunit	22q12.3	https://www.ncbi.nlm.nih.gov/gene/?term=1439+AND+homo+sapiens	1.93	**8.2E-08**	5.3E-06	PVRIG_High
3620	IDO1	indoleamine 2,3-dioxygenase 1	8p11.21	https://www.ncbi.nlm.nih.gov/gene/?term=3620+AND+homo+sapiens	1.99	**8.4E-08**	5.4E-06	PVRIG_High

5552	SRGN	serglycin	10q22.1	https://www.ncbi.nlm.nih.gov/gene/?term=5552+AND+homo+sapiens	2.15	**8.5E-08**	5.4E-06	PVRIG_High
6039	RNASE 6	ribonuclease A family member k6	14q11.2	https://www.ncbi.nlm.nih.gov/gene/?term=6039+AND+homo+sapiens	1.53	**1.0E-07**	6.5E-06	PVRIG_High
474344	GIMAP 6	GTPase, IMAP family member 6	7q36.1	https://www.ncbi.nlm.nih.gov/gene/?term=474344+AND+homo+sapiens	1.52	**1.1E-07**	6.8E-06	PVRIG_High
6355	CCL8	C-C motif chemokine ligand 8	17q12	https://www.ncbi.nlm.nih.gov/gene/?term=6355+AND+homo+sapiens	1.81	**1.3E-07**	7.8E-06	PVRIG_High
3113	HLA-DPA1	major histocompatibility complex, class II, DP alpha 1	6p21.32	https://www.ncbi.nlm.nih.gov/gene/?term=3113+AND+homo+sapiens	1.86	**1.4E-07**	8.6E-06	PVRIG_High
3109	HLA-DMB	major histocompatibility complex, class II, DM beta	6p21.32	https://www.ncbi.nlm.nih.gov/gene/?term=3109+AND+homo+sapiens	1.54	**1.5E-07**	8.9E-06	PVRIG_High
10346	TRIM22	tripartite motif containing 22	11p15.4	https://www.ncbi.nlm.nih.gov/gene/?term=10346+AND+homo+sapiens	1.78	**2.0E-07**	1.2E-05	PVRIG_High
10563	CXCL13	C-X-C motif chemokine ligand 13	4q21.1	https://www.ncbi.nlm.nih.gov/gene/?term=10563+AND+homo+sapiens	1.59	**2.2E-07**	1.3E-05	PVRIG_High
5996	RGS1	regulator of G protein signaling 1	1q31.2	https://www.ncbi.nlm.nih.gov/gene/?term=5996+AND+homo+sapiens	1.73	**2.2E-07**	1.3E-05	PVRIG_High
10261	IGSF6	immunoglobulin superfamily member 6	16p12.2	https://www.ncbi.nlm.nih.gov/gene/?term=10261+AND+homo+sapiens	1.56	**3.6E-07**	2.0E-05	PVRIG_High
7852	CXCR4	C-X-C motif chemokine receptor 4	2q22.1	https://www.ncbi.nlm.nih.gov/gene/?term=7852+AND+homo+sapiens	1.73	**3.8E-07**	2.1E-05	PVRIG_High
972	CD74	CD74 molecule	5q33.1	https://www.ncbi.nlm.nih.gov/gene/?term=972+AND+homo+sapiens	1.54	**5.8E-07**	3.1E-05	PVRIG_High

894	CCND2	cyclin D2	12p13.32	https://www.ncbi.nlm.nih.gov/gene/?term=894+AND+homo+sapiens	1.88	**5.9E-07**	3.1E-05	**PVRIG_High**
3600	IL15	interleukin 15	4q31.21	https://www.ncbi.nlm.nih.gov/gene/?term=3600+AND+homo+sapiens	1.51	**6.7E-07**	3.4E-05	**PVRIG_High**
9770	RASSF2	Ras association domain family member 2	20p13	https://www.ncbi.nlm.nih.gov/gene/?term=9770+AND+homo+sapiens	1.58	**8.5E-07**	4.3E-05	**PVRIG_High**
3627	CXCL10	C-X-C motif chemokine ligand 10	4q21.1	https://www.ncbi.nlm.nih.gov/gene/?term=3627+AND+homo+sapiens	2.38	**8.8E-07**	4.4E-05	**PVRIG_High**
6275	S100A4	S100 calcium binding protein A4	1q21.3	https://www.ncbi.nlm.nih.gov/gene/?term=6275+AND+homo+sapiens	2.03	**1.4E-06**	6.8E-05	**PVRIG_High**
64231	MS4A6A	membrane spanning 4-domains A6A	11q12.2	https://www.ncbi.nlm.nih.gov/gene/?term=64231+AND+homo+sapiens	1.60	**1.5E-06**	7.2E-05	**PVRIG_High**
10797	MTHFD2	methylenetetrahydrofolate dehydrogenase (NADP+ dependent) 2, methenyltetrahydrofolate cyclohydrolase	2p13.1	https://www.ncbi.nlm.nih.gov/gene/?term=10797+AND+homo+sapiens	2.10	**1.5E-06**	7.2E-05	**PVRIG_High**
5698	PSMB9	proteasome subunit beta 9	6p21.32	https://www.ncbi.nlm.nih.gov/gene/?term=5698+AND+homo+sapiens	1.73	**1.8E-06**	8.5E-05	**PVRIG_High**
5699	PSMB10	proteasome subunit beta 10	16q22.1	https://www.ncbi.nlm.nih.gov/gene/?term=5699+AND+homo+sapiens	1.64	**2.0E-06**	9.3E-05	**PVRIG_High**
2633	GBP1	guanylate binding protein 1	1p22.2	https://www.ncbi.nlm.nih.gov/gene/?term=2633+AND+homo+sapiens	1.87	**2.7E-06**	1.2E-04	**PVRIG_High**
9332	CD163	CD163 molecule	12p13.31	https://www.ncbi.nlm.nih.gov/gene/?term=9332+AND+homo+sapiens	1.62	**3.0E-06**	1.3E-04	**PVRIG_High**

3123	HLA-DRB1	major histocompatibility complex, class II, DR beta 1	6p21.32	https://www.ncbi.nlm.nih.gov/gene/?term=3123+AND+homo+sapiens	1.79	**3.8E-06**	1.6E-04	PVRIG_High
6362	CCL18	C-C motif chemokine ligand 18	17q12	https://www.ncbi.nlm.nih.gov/gene/?term=6362+AND+homo+sapiens	1.67	**4.7E-06**	2.0E-04	PVRIG_High
5272	SERPINB9	serpin family B member 9	6p25.2	https://www.ncbi.nlm.nih.gov/gene/?term=5272+AND+homo+sapiens	1.55	**9.3E-06**	3.6E-04	PVRIG_High
6347	CCL2	C-C motif chemokine ligand 2	17q12	https://www.ncbi.nlm.nih.gov/gene/?term=6347+AND+homo+sapiens	2.04	**1.4E-05**	5.1E-04	PVRIG_High
91316	GUSBP11	glucuronidase, beta pseudogene 11	22q11.23	https://www.ncbi.nlm.nih.gov/gene/?term=91316+AND+homo+sapiens	1.94	**1.6E-05**	5.5E-04	PVRIG_High
5920	RARRES3	retinoic acid receptor responder 3	11q12.3	https://www.ncbi.nlm.nih.gov/gene/?term=5920+AND+homo+sapiens	1.54	**1.9E-05**	6.5E-04	PVRIG_High
3965	LGALS9	galectin 9	17q11.2	https://www.ncbi.nlm.nih.gov/gene/?term=3965+AND+homo+sapiens	1.50	**2.4E-05**	7.9E-04	PVRIG_High
4332	MNDA	myeloid cell nuclear differentiation antigen	1q23.1	https://www.ncbi.nlm.nih.gov/gene/?term=4332+AND+homo+sapiens	1.53	**3.0E-05**	9.6E-04	PVRIG_High
8877	SPHK1	sphingosine kinase 1	17q25.1	https://www.ncbi.nlm.nih.gov/gene/?term=8877+AND+homo+sapiens	1.77	**3.6E-05**	1.1E-03	PVRIG_High
2207	FCER1G	Fc fragment of IgE receptor Ig	1q23.3	https://www.ncbi.nlm.nih.gov/gene/?term=2207+AND+homo+sapiens	1.51	**4.4E-05**	1.3E-03	PVRIG_High
8519	IFITM1	interferon induced transmembrane protein 1	11p15.5	https://www.ncbi.nlm.nih.gov/gene/?term=8519+AND+homo+sapiens	1.51	**4.9E-05**	1.4E-03	PVRIG_High
51338	MS4A4A	membrane spanning 4-domains A4A	11q12.2	https://www.ncbi.nlm.nih.gov/gene/?term=51338+AND+homo+sapiens	1.54	**5.3E-05**	1.6E-03	PVRIG_High

3117	HLA-DQA1	major histocompatibility complex, class II, DQ alpha 1	6p21.32	https://www.ncbi.nlm.nih.gov/gene/?term=3117+AND+homo+sapiens	2.81	**5.8E-05**	1.7E-03	PVRIG_High
10123	ARL4C	ADP ribosylation factor like GTPase 4C	2q37.1	https://www.ncbi.nlm.nih.gov/gene/?term=10123+AND+homo+sapiens	1.54	**5.9E-05**	1.7E-03	PVRIG_High
5360	PLTP	phospholipid transfer protein	20q13.12	https://www.ncbi.nlm.nih.gov/gene/?term=5360+AND+homo+sapiens	1.69	**7.4E-05**	2.1E-03	PVRIG_High
922	CD5L	CD5 molecule like	1q23.1	https://www.ncbi.nlm.nih.gov/gene/?term=922+AND+homo+sapiens	1.62	**7.5E-05**	2.1E-03	PVRIG_High
8826	IQGAP1	IQ motif containing GTPase activating protein 1	15q26.1	https://www.ncbi.nlm.nih.gov/gene/?term=8826+AND+homo+sapiens	1.52	**7.8E-05**	2.1E-03	PVRIG_High
11031	RAB31	RAB31, member RAS oncogene family	18p11.22	https://www.ncbi.nlm.nih.gov/gene/?term=11031+AND+homo+sapiens	1.55	**9.2E-05**	2.4E-03	PVRIG_High
597	BCL2A1	BCL2 related protein A1	15q25.1	https://www.ncbi.nlm.nih.gov/gene/?term=597+AND+homo+sapiens	1.55	**1.7E-04**	3.9E-03	PVRIG_High
8531	YBX3	Y-box binding protein 3	12p13.2	https://www.ncbi.nlm.nih.gov/gene/?term=8531+AND+homo+sapiens	1.79	**1.7E-04**	3.9E-03	PVRIG_High
10964	IFI44L	interferon induced protein 44 like	1p31.1	https://www.ncbi.nlm.nih.gov/gene/?term=10964+AND+homo+sapiens	2.04	**1.8E-04**	4.0E-03	PVRIG_High
6366	CCL21	C-C motif chemokine ligand 21	9p13.3	https://www.ncbi.nlm.nih.gov/gene/?term=6366+AND+homo+sapiens	1.54	**2.4E-04**	5.0E-03	PVRIG_High
5734	PTGER4	prostaglandin E receptor 4	5p13.1	https://www.ncbi.nlm.nih.gov/gene/?term=5734+AND+homo+sapiens	1.53	**2.6E-04**	5.2E-03	PVRIG_High
3371	TNC	tenascin C	9q33.1	https://www.ncbi.nlm.nih.gov/gene/?term=3371+AND+homo+sapiens	2.06	**2.7E-04**	5.4E-03	PVRIG_High
4599	MX1	MX dynamin like GTPase 1	21q22.3	https://www.ncbi.nlm.nih.gov/gene/?term=4599+AND+homo+sapiens	1.50	**2.8E-04**	5.5E-03	PVRIG_High
10457	GPNMB	glycoprotein nmb	7p15.3	https://www.ncbi.nlm.nih.gov/gene/?term=10457+AND+homo+sapiens	1.67	**3.8E-04**	6.8E-03	PVRIG_High

3122	HLA-DRA	major histocompatibility complex, class II, DR alpha	6p21.32	https://www.ncbi.nlm.nih.gov/gene/?term=3122+AND+homo+sapiens	1.79	**3.9E-04**	6.9E-03	**PVRIG_High**
29940	DSE	dermatan sulfate epimerase	6q22.1	https://www.ncbi.nlm.nih.gov/gene/?term=29940+AND+homo+sapiens	1.55	**4.7E-04**	7.9E-03	**PVRIG_High**
1520	CTSS	cathepsin S	1q21.3	https://www.ncbi.nlm.nih.gov/gene/?term=1520+AND+homo+sapiens	1.57	**5.9E-04**	9.3E-03	**PVRIG_High**
4321	MMP12	matrix metallopeptidase 12	11q22.2	https://www.ncbi.nlm.nih.gov/gene/?term=4321+AND+homo+sapiens	1.70	**6.9E-04**	1.0E-02	**PVRIG_High**
822	CAPG	capping actin protein, gelsolin like	2p11.2	https://www.ncbi.nlm.nih.gov/gene/?term=822+AND+homo+sapiens	1.64	**7.2E-04**	1.1E-02	**PVRIG_High**
26509	MYOF	myoferlin	10q23.33	https://www.ncbi.nlm.nih.gov/gene/?term=26509+AND+homo+sapiens	2.10	**9.2E-04**	1.3E-02	**PVRIG_High**
56925	LXN	latexin	3q25.32	https://www.ncbi.nlm.nih.gov/gene/?term=56925+AND+homo+sapiens	1.57	**1.4E-03**	1.7E-02	**PVRIG_High**
240	ALOX5	arachidonate 5-lipoxygenase	10q11.21	https://www.ncbi.nlm.nih.gov/gene/?term=240+AND+homo+sapiens	1.58	**1.5E-03**	1.7E-02	**PVRIG_High**
3428	IFI16	interferon gamma inducible protein 16	1q23.1	https://www.ncbi.nlm.nih.gov/gene/?term=3428+AND+homo+sapiens	1.57	**1.7E-03**	1.9E-02	**PVRIG_High**
8821	INPP4B	inositol polyphosphate-4-phosphatase type II B	4q31.21	https://www.ncbi.nlm.nih.gov/gene/?term=8821+AND+homo+sapiens	1.99	**2.1E-03**	2.2E-02	**PVRIG_High**
4053	LTBP2	latent transforming growth factor beta binding protein 2	14q24.3	https://www.ncbi.nlm.nih.gov/gene/?term=4053+AND+homo+sapiens	1.51	**2.6E-03**	2.5E-02	**PVRIG_High**
57823	SLAMF7	SLAM family member 7	1q23.3	https://www.ncbi.nlm.nih.gov/gene/?term=57823+AND+homo+sapiens	1.54	**2.6E-03**	2.5E-02	**PVRIG_High**
3223	HOXC6	homeobox C6	12q13.13	https://www.ncbi.nlm.nih.gov/gene/?term=3223+AND+homo+sapiens	1.70	**3.1E-03**	2.8E-02	**PVRIG_High**

9547	CXCL14	C-X-C motif chemokine ligand 14	5q31.1	https://www.ncbi.nlm.nih.gov/gene/?term=9547+AND+homo+sapiens	1.64	**3.6E-03**	3.1E-02	PVRIG_High
2950	GSTP1	glutathione S-transferase pi 1	11q13.2	https://www.ncbi.nlm.nih.gov/gene/?term=2950+AND+homo+sapiens	1.67	**3.6E-03**	3.1E-02	PVRIG_High
2171	FABP5	fatty acid binding protein 5	8q21.13	https://www.ncbi.nlm.nih.gov/gene/?term=2171+AND+homo+sapiens	1.59	**4.2E-03**	3.4E-02	PVRIG_High
10512	SEMA3C	semaphorin 3C	7q21.11	https://www.ncbi.nlm.nih.gov/gene/?term=10512+AND+homo+sapiens	1.86	**4.4E-03**	3.5E-02	PVRIG_High
6387	CXCL12	C-X-C motif chemokine ligand 12	10q11.21	https://www.ncbi.nlm.nih.gov/gene/?term=6387+AND+homo+sapiens	1.59	**4.8E-03**	3.7E-02	PVRIG_High
165	AEBP1	AE binding protein 1	7p13	https://www.ncbi.nlm.nih.gov/gene/?term=165+AND+homo+sapiens	1.71	**5.2E-03**	3.9E-02	PVRIG_High
10631	POSTN	periostin	13q13.3	https://www.ncbi.nlm.nih.gov/gene/?term=10631+AND+homo+sapiens	1.79	**5.9E-03**	4.3E-02	PVRIG_High
8740	TNFSF14	TNF superfamily member 14	19p13.3	https://www.ncbi.nlm.nih.gov/gene/?term=8740+AND+homo+sapiens	1.59	**6.3E-03**	4.5E-02	PVRIG_High
9023	CH25H	cholesterol 25-hydroxylase	10q23.31	https://www.ncbi.nlm.nih.gov/gene/?term=9023+AND+homo+sapiens	1.65	**6.5E-03**	4.5E-02	PVRIG_High
2669	GEM	GTP binding protein overexpressed in skeletal muscle	8q22.1	https://www.ncbi.nlm.nih.gov/gene/?term=2669+AND+homo+sapiens	2.25	**6.8E-03**	4.6E-02	PVRIG_High
3119	HLA-DQB1	major histocompatibility complex, class II, DQ beta 1	6p21.32	https://www.ncbi.nlm.nih.gov/gene/?term=3119+AND+homo+sapiens	1.67	**7.6E-03**	5.0E-02	PVRIG_High
1466	CSRP2	cysteine and glycine rich protein 2	12q21.2	https://www.ncbi.nlm.nih.gov/gene/?term=1466+AND+homo+sapiens	-1.58	**2.3E-05**	7.8E-04	PVRIG_Low
3303	HSPA1A	heat shock protein family A	6p21.33	https://www.ncbi.nlm.nih.gov/gene/?term=3303+AND+homo+sapiens	-1.55	**1.5E-04**	3.5E-03	PVRIG_Low

		(Hsp70) member 1A						
3290	HSD11B 1	hydroxysteroid 11-beta dehydrogenase 1	1q32.2	https://www.ncbi.nlm.nih.gov/gene/?term=3290+AND+homo+sapiens	-1.73	**5.2E-04**	8.5E-03	PVRIG_Low
1803	DPP4	dipeptidyl peptidase 4	2q24.2	https://www.ncbi.nlm.nih.gov/gene/?term=1803+AND+homo+sapiens	-1.56	**5.3E-04**	8.7E-03	PVRIG_Low
10681	GNB5	G protein subunit beta 5	15q21.2	https://www.ncbi.nlm.nih.gov/gene/?term=10681+AND+homo+sapiens	-1.69	**1.1E-03**	1.4E-02	PVRIG_Low
23460	ABCA6	ATP binding cassette subfamily A member 6	17q24.2-q24.3	https://www.ncbi.nlm.nih.gov/gene/?term=23460+AND+homo+sapiens	-1.50	**1.8E-03**	2.0E-02	PVRIG_Low
10050	SLC17A 4	solute carrier family 17 member 4	6p22.2	https://www.ncbi.nlm.nih.gov/gene/?term=10050+AND+homo+sapiens	-1.50	**2.0E-03**	2.1E-02	PVRIG_Low
23600	AMAC R	alpha-methylacyl-CoA racemase	5p13.2	https://www.ncbi.nlm.nih.gov/gene/?term=23600+AND+homo+sapiens	-1.59	**2.1E-03**	2.2E-02	PVRIG_Low
10882	C1QL1	complement C1q like 1	17q21.31	https://www.ncbi.nlm.nih.gov/gene/?term=10882+AND+homo+sapiens	-1.81	**2.6E-03**	2.5E-02	PVRIG_Low
5313	PKLR	pyruvate kinase L/R	1q22	https://www.ncbi.nlm.nih.gov/gene/?term=5313+AND+homo+sapiens	-1.52	**3.5E-03**	3.0E-02	PVRIG_Low
53841	CDHR5	cadherin related family member 5	11p15.5	https://www.ncbi.nlm.nih.gov/gene/?term=53841+AND+homo+sapiens	-1.51	**3.8E-03**	3.2E-02	PVRIG_Low
4311	MME	membrane metalloendopeptidase	3q25.2	https://www.ncbi.nlm.nih.gov/gene/?term=4311+AND+homo+sapiens	-2.37	**5.8E-03**	4.2E-02	PVRIG_Low
5756	TWF1	twinfilin actin binding protein 1	12q12	https://www.ncbi.nlm.nih.gov/gene/?term=5756+AND+homo+sapiens	-1.51	**6.1E-03**	4.4E-02	PVRIG_Low
1491	CTH	cystathionine gamma-lyase	1p31.1	https://www.ncbi.nlm.nih.gov/gene/?term=1491+AND+homo+sapiens	-1.56	**6.4E-03**	4.5E-02	PVRIG_Low
64388	GREM2	gremlin 2, DAN family BMP antagonist	1q43	https://www.ncbi.nlm.nih.gov/gene/?term=64388+AND+homo+sapiens	-3.01	**6.4E-03**	4.5E-02	PVRIG_Low

Table S4. Ontology analysis of the 181 genes differentially expressed between *PVRIG* High and Low groups in the learning set (GSE14520, n=242)

GO ID	Description	p-value	Adjusted p-value	Fold enrichment	GeneRatio	BgRatio
GO:0050900	leukocyte migration	1.99E-23	**4.83E-20**	8.85	35/153	478/18493
GO:0030595	leukocyte chemotaxis	6.31E-23	**7.65E-20**	14.48	26/153	217/18493
GO:0060326	cell chemotaxis	1.17E-20	**9.46E-18**	11.06	27/153	295/18493
GO:0042110	T cell activation	5.82E-20	**3.52E-17**	8.31	31/153	451/18493
GO:0007159	leukocyte cell-cell adhesion	1.73E-19	**8.36E-17**	9.98	27/153	327/18493
GO:0048247	lymphocyte chemotaxis	2.51E-18	**1.01E-15**	28.78	15/153	63/18493
GO:0072676	lymphocyte migration	1.42E-17	**4.90E-15**	19.57	17/153	105/18493
GO:0050863	regulation of T cell activation	6.39E-17	**1.93E-14**	9.51	24/153	305/18493
GO:0009615	response to virus	1.33E-16	**3.57E-14**	9.21	24/153	315/18493
GO:0070661	leukocyte proliferation	1.47E-16	**3.57E-14**	9.82	23/153	283/18493
GO:0030593	neutrophil chemotaxis	1.90E-16	**4.18E-14**	19.15	16/153	101/18493
GO:0034341	response to interferon-gamma	2.38E-16	**4.81E-14**	12.21	20/153	198/18493
GO:0097529	myeloid leukocyte migration	2.63E-16	**4.90E-14**	12.15	20/153	199/18493
GO:1903037	regulation of leukocyte cell-cell adhesion	3.16E-16	**5.46E-14**	9.49	23/153	293/18493
GO:0071346	cellular response to interferon-gamma	4.96E-16	**8.01E-14**	12.90	19/153	178/18493
GO:0002688	regulation of leukocyte chemotaxis	6.69E-16	**1.01E-13**	17.74	16/153	109/18493

GO:0097530	granulocyte migration	9.95E-16	**1.41E-13**	15.33	17/153	134/18493
GO:1990266	neutrophil migration	1.05E-15	**1.41E-13**	17.27	16/153	112/18493
GO:0070663	regulation of leukocyte proliferation	1.30E-15	**1.66E-13**	11.19	20/153	216/18493
GO:0051249	regulation of lymphocyte activation	1.86E-15	**2.25E-13**	6.91	27/153	472/18493
GO:0071621	granulocyte chemotaxis	3.22E-15	**3.72E-13**	16.12	16/153	120/18493
GO:0046651	lymphocyte proliferation	5.21E-15	**5.52E-13**	9.61	21/153	264/18493
GO:0050870	positive regulation of T cell activation	5.24E-15	**5.52E-13**	11.37	19/153	202/18493
GO:0032943	mononuclear cell proliferation	6.06E-15	**6.11E-13**	9.54	21/153	266/18493
GO:0002690	positive regulation of leukocyte chemotaxis	7.43E-15	**7.19E-13**	20.14	14/153	84/18493
GO:0002685	regulation of leukocyte migration	9.70E-15	**9.04E-13**	12.09	18/153	180/18493
GO:0050920	regulation of chemotaxis	1.07E-14	**9.63E-13**	10.94	19/153	210/18493
GO:0070098	chemokine-mediated signaling pathway	1.24E-14	**1.07E-12**	19.45	14/153	87/18493
GO:0022407	regulation of cell-cell adhesion	1.43E-14	**1.20E-12**	7.48	24/153	388/18493
GO:1903039	positive regulation of leukocyte cell-cell adhesion	1.52E-14	**1.23E-12**	10.73	19/153	214/18493
GO:0022409	positive regulation of cell-cell adhesion	2.35E-14	**1.84E-12**	9.63	20/153	251/18493
GO:1990868	response to chemokine	4.43E-14	**3.25E-12**	17.81	14/153	95/18493
GO:1990869	cellular response to chemokine	4.43E-14	**3.25E-12**	17.81	14/153	95/18493
GO:0002687	positive regulation of leukocyte migration	7.38E-14	**5.26E-12**	14.98	15/153	121/18493
GO:0050670	regulation of lymphocyte proliferation	9.46E-14	**6.54E-12**	10.61	18/153	205/18493
GO:0032944	regulation of mononuclear cell proliferation	1.03E-13	**6.90E-12**	10.56	18/153	206/18493
GO:0050867	positive regulation of cell activation	1.05E-13	**6.90E-12**	7.24	23/153	384/18493
GO:0042098	T cell proliferation	1.20E-13	**7.62E-12**	11.54	17/153	178/18493
GO:0045785	positive regulation of cell adhesion	2.12E-13	**1.32E-11**	7.00	23/153	397/18493
GO:1901623	regulation of lymphocyte chemotaxis	3.97E-13	**2.40E-11**	41.84	9/153	26/18493
GO:0002696	positive regulation of leukocyte activation	4.90E-13	**2.89E-11**	7.15	22/153	372/18493
GO:0032103	positive regulation of response to external stimulus	5.59E-13	**3.22E-11**	8.14	20/153	297/18493
GO:2000401	regulation of lymphocyte migration	1.72E-12	**9.69E-11**	22.53	11/153	59/18493
GO:0042129	regulation of T cell proliferation	2.42E-12	**1.33E-10**	11.85	15/153	153/18493
GO:0050921	positive regulation of chemotaxis	3.70E-12	**1.99E-10**	13.02	14/153	130/18493
GO:0002548	monocyte chemotaxis	4.41E-12	**2.32E-10**	20.77	11/153	64/18493

GO:0001819	positive regulation of cytokine production	6.00E-12	**3.09E-10**	6.30	22/153	422/18493
GO:0051607	defense response to virus	6.76E-12	**3.41E-10**	9.01	17/153	228/18493
GO:2000403	positive regulation of lymphocyte migration	1.11E-11	**5.51E-10**	30.22	9/153	36/18493
GO:0050852	T cell receptor signaling pathway	2.63E-11	**1.27E-09**	11.28	14/153	150/18493
GO:0051251	positive regulation of lymphocyte activation	3.98E-11	**1.89E-09**	6.90	19/153	333/18493
GO:0072678	T cell migration	4.96E-11	**2.31E-09**	20.49	10/153	59/18493
GO:0002697	regulation of immune effector process	9.38E-11	**4.28E-09**	5.78	21/153	439/18493
GO:0030217	T cell differentiation	1.06E-10	**4.78E-09**	8.26	16/153	234/18493
GO:0071674	mononuclear cell migration	1.09E-10	**4.81E-09**	15.64	11/153	85/18493
GO:0071356	cellular response to tumor necrosis factor	1.13E-10	**4.91E-09**	8.23	16/153	235/18493
GO:0035747	natural killer cell chemotaxis	1.30E-10	**5.51E-09**	65.93	6/153	11/18493
GO:2000106	regulation of leukocyte apoptotic process	1.41E-10	**5.90E-09**	15.28	11/153	87/18493
GO:0019882	antigen processing and presentation	2.09E-10	**8.58E-09**	9.67	14/153	175/18493
GO:2000107	negative regulation of leukocyte apoptotic process	3.24E-10	**1.31E-08**	21.33	9/153	51/18493
GO:0034612	response to tumor necrosis factor	4.26E-10	**1.69E-08**	7.52	16/153	257/18493
GO:0042102	positive regulation of T cell proliferation	4.67E-10	**1.83E-08**	13.71	11/153	97/18493
GO:0050851	antigen receptor-mediated signaling pathway	4.77E-10	**1.83E-08**	7.47	16/153	259/18493
GO:0002683	negative regulation of immune system process	5.90E-10	**2.23E-08**	5.53	20/153	437/18493
GO:0042119	neutrophil activation	9.32E-10	**3.47E-08**	5.10	21/153	498/18493
GO:0050671	positive regulation of lymphocyte proliferation	9.91E-10	**3.64E-08**	10.99	12/153	132/18493
GO:0032946	positive regulation of mononuclear cell proliferation	1.08E-09	**3.91E-08**	10.91	12/153	133/18493
GO:0071887	leukocyte apoptotic process	1.36E-09	**4.83E-08**	12.43	11/153	107/18493
GO:0002429	immune response-activating cell surface receptor signaling pathway	1.57E-09	**5.51E-08**	5.55	19/153	414/18493
GO:0010818	T cell chemotaxis	1.79E-09	**6.14E-08**	31.34	7/153	27/18493
GO:0070665	positive regulation of leukocyte proliferation	1.80E-09	**6.14E-08**	10.43	12/153	139/18493
GO:0043312	neutrophil degranulation	3.55E-09	**1.19E-07**	4.98	20/153	485/18493
GO:0060333	interferon-gamma-mediated signaling pathway	3.63E-09	**1.20E-07**	13.43	10/153	90/18493
GO:0002283	neutrophil activation involved in immune response	3.94E-09	**1.28E-07**	4.95	20/153	488/18493
GO:2000406	positive regulation of T cell migration	4.01E-09	**1.28E-07**	28.20	7/153	30/18493

GO:0002449	lymphocyte mediated immunity	4.02E-09	**1.28E-07**	5.97	17/153	344/18493
GO:0002768	immune response-regulating cell surface receptor signaling pathway	5.13E-09	**1.61E-07**	5.16	19/153	445/18493
GO:1902105	regulation of leukocyte differentiation	5.65E-09	**1.75E-07**	6.84	15/153	265/18493
GO:0002446	neutrophil mediated immunity	5.75E-09	**1.76E-07**	4.84	20/153	499/18493
GO:0002478	antigen processing and presentation of exogenous peptide antigen	7.78E-09	**2.35E-07**	10.55	11/153	126/18493
GO:0019886	antigen processing and presentation of exogenous peptide antigen via MHC class II	8.40E-09	**2.51E-07**	12.33	10/153	98/18493
GO:0140131	positive regulation of lymphocyte chemotaxis	1.02E-08	**3.02E-07**	36.26	6/153	20/18493
GO:0002495	antigen processing and presentation of peptide antigen via MHC class II	1.13E-08	**3.29E-07**	11.97	10/153	101/18493
GO:0002504	antigen processing and presentation of peptide or polysaccharide antigen via MHC class II	1.24E-08	**3.58E-07**	11.85	10/153	102/18493
GO:0045123	cellular extravasation	1.38E-08	**3.92E-07**	17.91	8/153	54/18493
GO:0019884	antigen processing and presentation of exogenous antigen	1.49E-08	**4.19E-07**	9.92	11/153	134/18493
GO:0001909	leukocyte mediated cytotoxicity	1.65E-08	**4.58E-07**	11.51	10/153	105/18493
GO:0048002	antigen processing and presentation of peptide antigen	2.18E-08	**6.00E-07**	9.57	11/153	139/18493
GO:0070371	ERK1 and ERK2 cascade	2.45E-08	**6.66E-07**	5.67	16/153	341/18493
GO:0002407	dendritic cell chemotaxis	2.61E-08	**7.03E-07**	31.53	6/153	23/18493
GO:1902107	positive regulation of leukocyte differentiation	2.73E-08	**7.25E-07**	9.36	11/153	142/18493
GO:0030098	lymphocyte differentiation	2.77E-08	**7.28E-07**	5.62	16/153	344/18493
GO:2000404	regulation of T cell migration	3.43E-08	**8.90E-07**	21.15	7/153	40/18493
GO:0030101	natural killer cell activation	3.46E-08	**8.90E-07**	12.80	9/153	85/18493
GO:1903708	positive regulation of hemopoiesis	4.03E-08	**1.03E-06**	7.93	12/153	183/18493
GO:0036336	dendritic cell migration	5.84E-08	**1.47E-06**	27.89	6/153	26/18493
GO:1903706	regulation of hemopoiesis	7.04E-08	**1.76E-06**	4.92	17/153	418/18493
GO:0007162	negative regulation of cell adhesion	7.36E-08	**1.82E-06**	6.13	14/153	276/18493
GO:0070372	regulation of ERK1 and ERK2 cascade	8.18E-08	**2.00E-06**	5.60	15/153	324/18493
GO:0032680	regulation of tumor necrosis factor production	1.02E-07	**2.48E-06**	9.52	10/153	127/18493
GO:0032640	tumor necrosis factor production	1.18E-07	**2.84E-06**	9.37	10/153	129/18493

GO:0070374	positive regulation of ERK1 and ERK2 cascade	1.31E-07	**3.10E-06**	6.41	13/153	245/18493
GO:1903555	regulation of tumor necrosis factor superfamily cytokine production	1.37E-07	**3.22E-06**	9.23	10/153	131/18493
GO:0010819	regulation of T cell chemotaxis	1.47E-07	**3.43E-06**	37.77	5/153	16/18493
GO:0032760	positive regulation of tumor necrosis factor production	1.56E-07	**3.59E-06**	13.25	8/153	73/18493
GO:0002699	positive regulation of immune effector process	1.73E-07	**3.95E-06**	6.94	12/153	209/18493
GO:0071706	tumor necrosis factor superfamily cytokine production	1.82E-07	**4.12E-06**	8.95	10/153	135/18493
GO:0006874	cellular calcium ion homeostasis	1.89E-07	**4.24E-06**	4.59	17/153	448/18493
GO:0045580	regulation of T cell differentiation	2.09E-07	**4.64E-06**	8.82	10/153	137/18493
GO:1903557	positive regulation of tumor necrosis factor superfamily cytokine production	2.14E-07	**4.71E-06**	12.72	8/153	76/18493
GO:0055074	calcium ion homeostasis	2.83E-07	**6.17E-06**	4.46	17/153	461/18493
GO:0090026	positive regulation of monocyte chemotaxis	2.85E-07	**6.17E-06**	33.57	5/153	18/18493
GO:0006816	calcium ion transport	3.36E-07	**7.21E-06**	4.68	16/153	413/18493
GO:0042267	natural killer cell mediated cytotoxicity	4.88E-07	**1.04E-05**	14.59	7/153	58/18493
GO:0072503	cellular divalent inorganic cation homeostasis	5.43E-07	**1.14E-05**	4.25	17/153	483/18493
GO:0033622	integrin activation	6.64E-07	**1.39E-05**	28.78	5/153	21/18493
GO:0002573	myeloid leukocyte differentiation	7.58E-07	**1.57E-05**	6.75	11/153	197/18493
GO:0002228	natural killer cell mediated immunity	7.77E-07	**1.59E-05**	13.65	7/153	62/18493
GO:0001906	cell killing	7.85E-07	**1.60E-05**	7.65	10/153	158/18493
GO:0071677	positive regulation of mononuclear cell migration	8.53E-07	**1.72E-05**	27.47	5/153	22/18493
GO:0032729	positive regulation of interferon-gamma production	8.68E-07	**1.74E-05**	13.43	7/153	63/18493
GO:0097048	dendritic cell apoptotic process	9.10E-07	**1.79E-05**	48.35	4/153	10/18493
GO:2000668	regulation of dendritic cell apoptotic process	9.10E-07	**1.79E-05**	48.35	4/153	10/18493
GO:0045621	positive regulation of lymphocyte differentiation	1.11E-06	**2.18E-05**	10.29	8/153	94/18493
GO:0045619	regulation of lymphocyte differentiation	1.23E-06	**2.39E-05**	7.28	10/153	166/18493
GO:0070838	divalent metal ion transport	1.36E-06	**2.59E-05**	4.21	16/153	459/18493
GO:0090025	regulation of monocyte chemotaxis	1.36E-06	**2.59E-05**	25.18	5/153	24/18493
GO:0050663	cytokine secretion	1.42E-06	**2.69E-05**	6.33	11/153	210/18493
GO:0072511	divalent inorganic cation transport	1.48E-06	**2.77E-05**	4.19	16/153	462/18493

GO:0072677	eosinophil migration	1.69E-06	**3.14E-05**	24.17	5/153	25/18493
GO:0007204	positive regulation of cytosolic calcium ion concentration	1.82E-06	**3.36E-05**	5.09	13/153	309/18493
GO:0002448	mast cell mediated immunity	2.07E-06	**3.80E-05**	15.77	6/153	46/18493
GO:0045088	regulation of innate immune response	2.13E-06	**3.88E-05**	4.64	14/153	365/18493
GO:0043900	regulation of multi-organism process	2.58E-06	**4.66E-05**	4.56	14/153	371/18493
GO:0031349	positive regulation of defense response	2.69E-06	**4.82E-05**	4.25	15/153	427/18493
GO:0032496	response to lipopolysaccharide	3.06E-06	**5.45E-05**	4.85	13/153	324/18493
GO:0050707	regulation of cytokine secretion	3.13E-06	**5.53E-05**	6.57	10/153	184/18493
GO:0002237	response to molecule of bacterial origin	4.70E-06	**8.25E-05**	4.66	13/153	337/18493
GO:0070555	response to interleukin-1	4.92E-06	**8.57E-05**	7.16	9/153	152/18493
GO:0051209	release of sequestered calcium ion into cytosol	5.46E-06	**9.36E-05**	8.34	8/153	116/18493
GO:0051928	positive regulation of calcium ion transport	5.46E-06	**9.36E-05**	8.34	8/153	116/18493
GO:0002703	regulation of leukocyte mediated immunity	5.49E-06	**9.36E-05**	6.17	10/153	196/18493
GO:0045582	positive regulation of T cell differentiation	5.67E-06	**9.57E-05**	10.19	7/153	83/18493
GO:0001911	negative regulation of leukocyte mediated cytotoxicity	5.73E-06	**9.57E-05**	32.23	4/153	15/18493
GO:0010820	positive regulation of T cell chemotaxis	5.73E-06	**9.57E-05**	32.23	4/153	15/18493
GO:0032735	positive regulation of interleukin-12 production	6.10E-06	**1.01E-04**	18.89	5/153	32/18493
GO:0051283	negative regulation of sequestering of calcium ion	6.20E-06	**1.02E-04**	8.19	8/153	118/18493
GO:0002819	regulation of adaptive immune response	6.40E-06	**1.05E-04**	6.93	9/153	157/18493
GO:0006959	humoral immune response	6.86E-06	**1.12E-04**	4.50	13/153	349/18493
GO:0051282	regulation of sequestering of calcium ion	7.02E-06	**1.12E-04**	8.06	8/153	120/18493
GO:0043547	positive regulation of GTPase activity	7.06E-06	**1.12E-04**	4.18	14/153	405/18493
GO:0048872	homeostasis of number of cells	7.06E-06	**1.12E-04**	5.36	11/153	248/18493
GO:0051480	regulation of cytosolic calcium ion concentration	7.08E-06	**1.12E-04**	4.49	13/153	350/18493
GO:0045730	respiratory burst	7.15E-06	**1.12E-04**	18.31	5/153	33/18493
GO:0045576	mast cell activation	7.45E-06	**1.16E-04**	12.72	6/153	57/18493
GO:0031341	regulation of cell killing	8.38E-06	**1.29E-04**	9.61	7/153	88/18493
GO:0050715	positive regulation of cytokine secretion	8.42E-06	**1.29E-04**	7.86	8/153	123/18493
GO:0051208	sequestering of calcium ion	8.42E-06	**1.29E-04**	7.86	8/153	123/18493
GO:0048525	negative regulation of viral process	1.05E-05	**1.59E-04**	9.30	7/153	91/18493

GO:0006968	cellular defense response	1.11E-05	**1.67E-04**	11.89	6/153	61/18493
GO:0050691	regulation of defense response to virus by host	1.11E-05	**1.67E-04**	16.79	5/153	36/18493
GO:0043087	regulation of GTPase activity	1.13E-05	**1.68E-04**	3.77	15/153	481/18493
GO:0050922	negative regulation of chemotaxis	1.22E-05	**1.81E-04**	11.70	6/153	62/18493
GO:0031342	negative regulation of cell killing	1.26E-05	**1.86E-04**	26.86	4/153	18/18493
GO:0071347	cellular response to interleukin-1	1.27E-05	**1.86E-04**	7.44	8/153	130/18493
GO:0032649	regulation of interferon-gamma production	1.39E-05	**2.03E-04**	8.91	7/153	95/18493
GO:0097553	calcium ion transmembrane import into cytosol	1.41E-05	**2.05E-04**	7.33	8/153	132/18493
GO:0032651	regulation of interleukin-1 beta production	1.47E-05	**2.12E-04**	11.33	6/153	64/18493
GO:0050792	regulation of viral process	1.68E-05	**2.41E-04**	6.15	9/153	177/18493
GO:0071496	cellular response to external stimulus	1.87E-05	**2.67E-04**	4.42	12/153	328/18493
GO:0007520	myoblast fusion	1.89E-05	**2.68E-04**	15.11	5/153	40/18493
GO:1901739	regulation of myoblast fusion	1.97E-05	**2.77E-04**	24.17	4/153	20/18493
GO:0071622	regulation of granulocyte chemotaxis	2.41E-05	**3.34E-04**	14.39	5/153	42/18493
GO:0071675	regulation of mononuclear cell migration	2.41E-05	**3.34E-04**	14.39	5/153	42/18493
GO:0048245	eosinophil chemotaxis	2.42E-05	**3.34E-04**	23.02	4/153	21/18493
GO:0032609	interferon-gamma production	2.84E-05	**3.91E-04**	7.98	7/153	106/18493
GO:0001779	natural killer cell differentiation	2.93E-05	**4.00E-04**	21.98	4/153	22/18493
GO:0002706	regulation of lymphocyte mediated immunity	2.94E-05	**4.00E-04**	6.62	8/153	146/18493
GO:0043303	mast cell degranulation	3.04E-05	**4.11E-04**	13.74	5/153	44/18493
GO:0051924	regulation of calcium ion transport	3.08E-05	**4.14E-04**	5.06	10/153	239/18493
GO:0032611	interleukin-1 beta production	3.13E-05	**4.19E-04**	9.93	6/153	73/18493
GO:0030099	myeloid cell differentiation	3.30E-05	**4.39E-04**	3.88	13/153	405/18493
GO:0002279	mast cell activation involved in immune response	3.40E-05	**4.45E-04**	13.43	5/153	45/18493
GO:0002686	negative regulation of leukocyte migration	3.40E-05	**4.45E-04**	13.43	5/153	45/18493
GO:0060402	calcium ion transport into cytosol	3.40E-05	**4.45E-04**	6.49	8/153	149/18493
GO:0090023	positive regulation of neutrophil chemotaxis	3.53E-05	**4.60E-04**	21.02	4/153	23/18493
GO:0070664	negative regulation of leukocyte proliferation	3.66E-05	**4.74E-04**	9.67	6/153	75/18493
GO:0001910	regulation of leukocyte mediated cytotoxicity	3.94E-05	**5.07E-04**	9.54	6/153	76/18493

GO:0002460	adaptive immune response based on somatic recombination of immune receptors built from immunoglobulin superfamily domains	3.96E-05	**5.07E-04**	4.10	12/153	354/18493
GO:0032652	regulation of interleukin-1 production	4.25E-05	**5.41E-04**	9.42	6/153	77/18493
GO:0043903	regulation of symbiosis, encompassing mutualism through parasitism	4.95E-05	**6.25E-04**	5.36	9/153	203/18493
GO:0050901	leukocyte tethering or rolling	4.98E-05	**6.25E-04**	19.34	4/153	25/18493
GO:0071624	positive regulation of granulocyte chemotaxis	4.98E-05	**6.25E-04**	19.34	4/153	25/18493
GO:0045089	positive regulation of innate immune response	5.40E-05	**6.72E-04**	4.30	11/153	309/18493
GO:0050727	regulation of inflammatory response	5.41E-05	**6.72E-04**	3.70	13/153	425/18493
GO:0008154	actin polymerization or depolymerization	5.54E-05	**6.85E-04**	5.28	9/153	206/18493
GO:0032655	regulation of interleukin-12 production	5.70E-05	**7.00E-04**	12.09	5/153	50/18493
GO:0060142	regulation of syncytium formation by plasma membrane fusion	5.84E-05	**7.11E-04**	18.60	4/153	26/18493
GO:1902624	positive regulation of neutrophil migration	5.84E-05	**7.11E-04**	18.60	4/153	26/18493
GO:0060401	cytosolic calcium ion transport	5.89E-05	**7.14E-04**	6.01	8/153	161/18493
GO:0002468	dendritic cell antigen processing and presentation	6.39E-05	**7.66E-04**	36.26	3/153	10/18493
GO:1902713	regulation of interferon-gamma secretion	6.39E-05	**7.66E-04**	36.26	3/153	10/18493
GO:0002695	negative regulation of leukocyte activation	6.71E-05	**8.01E-04**	5.90	8/153	164/18493
GO:0032615	interleukin-12 production	6.90E-05	**8.19E-04**	11.62	5/153	52/18493
GO:1903038	negative regulation of leukocyte cell-cell adhesion	7.00E-05	**8.26E-04**	6.94	7/153	122/18493
GO:0000768	syncytium formation by plasma membrane fusion	7.56E-05	**8.81E-04**	11.40	5/153	53/18493
GO:0045071	negative regulation of viral genome replication	7.56E-05	**8.81E-04**	11.40	5/153	53/18493
GO:0140253	cell-cell fusion	7.56E-05	**8.81E-04**	11.40	5/153	53/18493
GO:0035456	response to interferon-beta	7.90E-05	**9.07E-04**	17.27	4/153	28/18493
GO:0090022	regulation of neutrophil chemotaxis	7.90E-05	**9.07E-04**	17.27	4/153	28/18493
GO:2000108	positive regulation of leukocyte apoptotic process	7.90E-05	**9.07E-04**	17.27	4/153	28/18493
GO:0031295	T cell costimulation	8.28E-05	**9.42E-04**	11.19	5/153	54/18493
GO:0043901	negative regulation of multi-organism process	8.29E-05	**9.42E-04**	5.72	8/153	169/18493
GO:0032612	interleukin-1 production	8.45E-05	**9.57E-04**	8.34	6/153	87/18493

GO:0001776	leukocyte homeostasis	9.01E-05	**1.01E-03**	8.24	6/153	88/18493
GO:0006949	syncytium formation	9.05E-05	**1.01E-03**	10.99	5/153	55/18493
GO:0031294	lymphocyte costimulation	9.05E-05	**1.01E-03**	10.99	5/153	55/18493
GO:0050854	regulation of antigen receptor-mediated signaling pathway	1.07E-04	**1.19E-03**	10.60	5/153	57/18493
GO:0070228	regulation of lymphocyte apoptotic process	1.07E-04	**1.19E-03**	10.60	5/153	57/18493
GO:0042100	B cell proliferation	1.09E-04	**1.20E-03**	7.97	6/153	91/18493
GO:0050777	negative regulation of immune response	1.10E-04	**1.20E-03**	6.46	7/153	131/18493
GO:0043122	regulation of I-kappaB kinase/NF-kappaB signaling	1.13E-04	**1.23E-03**	4.81	9/153	226/18493
GO:0033623	regulation of integrin activation	1.16E-04	**1.26E-03**	30.22	3/153	12/18493
GO:0008064	regulation of actin polymerization or depolymerization	1.19E-04	**1.28E-03**	5.43	8/153	178/18493
GO:0034694	response to prostaglandin	1.19E-04	**1.28E-03**	15.60	4/153	31/18493
GO:1902622	regulation of neutrophil migration	1.19E-04	**1.28E-03**	15.60	4/153	31/18493
GO:0030832	regulation of actin filament length	1.24E-04	**1.32E-03**	5.40	8/153	179/18493
GO:0046631	alpha-beta T cell activation	1.26E-04	**1.34E-03**	6.31	7/153	134/18493
GO:0030888	regulation of B cell proliferation	1.27E-04	**1.34E-03**	10.24	5/153	59/18493
GO:0002791	regulation of peptide secretion	1.33E-04	**1.40E-03**	3.38	13/153	465/18493
GO:0046633	alpha-beta T cell proliferation	1.35E-04	**1.42E-03**	15.11	4/153	32/18493
GO:2000116	regulation of cysteine-type endopeptidase activity	1.37E-04	**1.43E-03**	4.69	9/153	232/18493
GO:0051250	negative regulation of lymphocyte activation	1.45E-04	**1.51E-03**	6.18	7/153	137/18493
GO:0001771	immunological synapse formation	1.49E-04	**1.52E-03**	27.89	3/153	13/18493
GO:0035723	interleukin-15-mediated signaling pathway	1.49E-04	**1.52E-03**	27.89	3/153	13/18493
GO:0045059	positive thymic T cell selection	1.49E-04	**1.52E-03**	27.89	3/153	13/18493
GO:0071350	cellular response to interleukin-15	1.49E-04	**1.52E-03**	27.89	3/153	13/18493
GO:0050866	negative regulation of cell activation	1.50E-04	**1.52E-03**	5.26	8/153	184/18493
GO:0019058	viral life cycle	1.50E-04	**1.52E-03**	4.18	10/153	289/18493
GO:0070229	negative regulation of lymphocyte apoptotic process	1.53E-04	**1.54E-03**	14.65	4/153	33/18493
GO:1903900	regulation of viral life cycle	1.66E-04	**1.67E-03**	6.04	7/153	140/18493
GO:0052548	regulation of endopeptidase activity	1.86E-04	**1.86E-03**	3.48	12/153	417/18493
GO:0070234	positive regulation of T cell apoptotic process	1.89E-04	**1.88E-03**	25.90	3/153	14/18493

GO:0070672	response to interleukin-15	1.89E-04	**1.88E-03**	25.90	3/153	14/18493
GO:0002707	negative regulation of lymphocyte mediated immunity	1.93E-04	**1.91E-03**	13.81	4/153	35/18493
GO:0032418	lysosome localization	2.01E-04	**1.98E-03**	9.30	5/153	65/18493
GO:0070232	regulation of T cell apoptotic process	2.16E-04	**2.12E-03**	13.43	4/153	36/18493
GO:0061756	leukocyte adhesion to vascular endothelial cell	2.41E-04	**2.35E-03**	13.07	4/153	37/18493
GO:0050688	regulation of defense response to virus	2.49E-04	**2.42E-03**	8.89	5/153	68/18493
GO:0031348	negative regulation of defense response	2.55E-04	**2.46E-03**	4.86	8/153	199/18493
GO:0071222	cellular response to lipopolysaccharide	2.55E-04	**2.46E-03**	4.86	8/153	199/18493
GO:0097696	STAT cascade	2.64E-04	**2.54E-03**	5.60	7/153	151/18493
GO:0033077	T cell differentiation in thymus	2.66E-04	**2.55E-03**	8.76	5/153	69/18493
GO:0032731	positive regulation of interleukin-1 beta production	2.67E-04	**2.55E-03**	12.72	4/153	38/18493
GO:1903532	positive regulation of secretion by cell	2.81E-04	**2.67E-03**	3.56	11/153	373/18493
GO:1904951	positive regulation of establishment of protein localization	2.86E-04	**2.70E-03**	3.32	12/153	437/18493
GO:0001818	negative regulation of cytokine production	2.86E-04	**2.70E-03**	4.25	9/153	256/18493
GO:0051271	negative regulation of cellular component movement	2.88E-04	**2.70E-03**	3.55	11/153	374/18493
GO:0050708	regulation of protein secretion	2.92E-04	**2.73E-03**	3.31	12/153	438/18493
GO:0019932	second-messenger-mediated signaling	2.98E-04	**2.77E-03**	3.30	12/153	439/18493
GO:0009612	response to mechanical stimulus	3.02E-04	**2.79E-03**	4.74	8/153	204/18493
GO:0007249	I-kappaB kinase/NF-kappaB signaling	3.03E-04	**2.79E-03**	4.22	9/153	258/18493
GO:0031668	cellular response to extracellular stimulus	3.03E-04	**2.79E-03**	4.22	9/153	258/18493
GO:0061844	antimicrobial humoral immune response mediated by antimicrobial peptide	3.05E-04	**2.79E-03**	8.51	5/153	71/18493
GO:0001959	regulation of cytokine-mediated signaling pathway	3.09E-04	**2.83E-03**	5.46	7/153	155/18493
GO:0031334	positive regulation of protein complex assembly	3.12E-04	**2.84E-03**	4.20	9/153	259/18493
GO:0052547	regulation of peptidase activity	3.17E-04	**2.87E-03**	3.28	12/153	442/18493
GO:0043270	positive regulation of ion transport	3.21E-04	**2.89E-03**	4.18	9/153	260/18493
GO:0110053	regulation of actin filament organization	3.21E-04	**2.89E-03**	4.18	9/153	260/18493
GO:0071219	cellular response to molecule of bacterial origin	3.22E-04	**2.89E-03**	4.69	8/153	206/18493
GO:0070227	lymphocyte apoptotic process	3.25E-04	**2.91E-03**	8.39	5/153	72/18493

GO:0002577	regulation of antigen processing and presentation	3.47E-04	**3.07E-03**	21.33	3/153	17/18493
GO:0048535	lymph node development	3.47E-04	**3.07E-03**	21.33	3/153	17/18493
GO:0072643	interferon-gamma secretion	3.47E-04	**3.07E-03**	21.33	3/153	17/18493
GO:0002761	regulation of myeloid leukocyte differentiation	3.72E-04	**3.28E-03**	6.36	6/153	114/18493
GO:0030336	negative regulation of cell migration	3.74E-04	**3.28E-03**	3.73	10/153	324/18493
GO:0040013	negative regulation of locomotion	3.76E-04	**3.29E-03**	3.44	11/153	386/18493
GO:0043254	regulation of protein complex assembly	3.80E-04	**3.31E-03**	3.22	12/153	451/18493
GO:0018108	peptidyl-tyrosine phosphorylation	3.84E-04	**3.34E-03**	3.44	11/153	387/18493
GO:0042269	regulation of natural killer cell mediated cytotoxicity	3.95E-04	**3.40E-03**	11.51	4/153	42/18493
GO:0043300	regulation of leukocyte degranulation	3.95E-04	**3.40E-03**	11.51	4/153	42/18493
GO:0018212	peptidyl-tyrosine modification	4.10E-04	**3.49E-03**	3.41	11/153	390/18493
GO:0002544	chronic inflammatory response	4.14E-04	**3.49E-03**	20.14	3/153	18/18493
GO:0051767	nitric-oxide synthase biosynthetic process	4.14E-04	**3.49E-03**	20.14	3/153	18/18493
GO:0051769	regulation of nitric-oxide synthase biosynthetic process	4.14E-04	**3.49E-03**	20.14	3/153	18/18493
GO:0070230	positive regulation of lymphocyte apoptotic process	4.14E-04	**3.49E-03**	20.14	3/153	18/18493
GO:1901741	positive regulation of myoblast fusion	4.14E-04	**3.49E-03**	20.14	3/153	18/18493
GO:0019079	viral genome replication	4.27E-04	**3.59E-03**	6.20	6/153	117/18493
GO:0032956	regulation of actin cytoskeleton organization	4.42E-04	**3.69E-03**	3.65	10/153	331/18493
GO:0007584	response to nutrient	4.42E-04	**3.69E-03**	4.48	8/153	216/18493
GO:1903901	negative regulation of viral life cycle	4.44E-04	**3.70E-03**	7.85	5/153	77/18493
GO:0019730	antimicrobial humoral response	4.47E-04	**3.71E-03**	6.15	6/153	118/18493
GO:0060759	regulation of response to cytokine stimulus	4.51E-04	**3.73E-03**	5.13	7/153	165/18493
GO:0033209	tumor necrosis factor-mediated signaling pathway	4.68E-04	**3.85E-03**	6.09	6/153	119/18493
GO:0002715	regulation of natural killer cell mediated immunity	4.73E-04	**3.88E-03**	10.99	4/153	44/18493
GO:0007015	actin filament organization	4.76E-04	**3.90E-03**	3.35	11/153	397/18493
GO:0010560	positive regulation of glycoprotein biosynthetic process	4.88E-04	**3.98E-03**	19.08	3/153	19/18493
GO:0002704	negative regulation of leukocyte mediated immunity	5.16E-04	**4.15E-03**	10.74	4/153	45/18493
GO:0014911	positive regulation of smooth muscle cell migration	5.16E-04	**4.15E-03**	10.74	4/153	45/18493
GO:0032653	regulation of interleukin-10 production	5.16E-04	**4.15E-03**	10.74	4/153	45/18493
GO:0032732	positive regulation of interleukin-1 production	5.16E-04	**4.15E-03**	10.74	4/153	45/18493

GO:0031589	cell-substrate adhesion	5.20E-04	**4.17E-03**	3.58	10/153	338/18493
GO:0051222	positive regulation of protein transport	5.29E-04	**4.22E-03**	3.31	11/153	402/18493
GO:0042116	macrophage activation	5.30E-04	**4.22E-03**	7.55	5/153	80/18493
GO:2000146	negative regulation of cell motility	5.32E-04	**4.22E-03**	3.57	10/153	339/18493
GO:0022408	negative regulation of cell-cell adhesion	5.40E-04	**4.27E-03**	4.98	7/153	170/18493
GO:0051047	positive regulation of secretion	5.51E-04	**4.34E-03**	3.29	11/153	404/18493
GO:0031952	regulation of protein autophosphorylation	5.61E-04	**4.40E-03**	10.51	4/153	46/18493
GO:0045824	negative regulation of innate immune response	5.61E-04	**4.40E-03**	10.51	4/153	46/18493
GO:0033630	positive regulation of cell adhesion mediated by integrin	5.71E-04	**4.45E-03**	18.13	3/153	20/18493
GO:0035458	cellular response to interferon-beta	5.71E-04	**4.45E-03**	18.13	3/153	20/18493
GO:1902903	regulation of supramolecular fiber organization	5.83E-04	**4.52E-03**	3.52	10/153	343/18493
GO:0032613	interleukin-10 production	6.09E-04	**4.70E-03**	10.29	4/153	47/18493
GO:0150076	neuroinflammatory response	6.09E-04	**4.70E-03**	10.29	4/153	47/18493
GO:0002285	lymphocyte activation involved in immune response	6.20E-04	**4.76E-03**	4.86	7/153	174/18493
GO:0014910	regulation of smooth muscle cell migration	6.27E-04	**4.81E-03**	7.28	5/153	83/18493
GO:0071216	cellular response to biotic stimulus	6.50E-04	**4.97E-03**	4.22	8/153	229/18493
GO:0009409	response to cold	6.60E-04	**4.97E-03**	10.07	4/153	48/18493
GO:0045058	T cell selection	6.60E-04	**4.97E-03**	10.07	4/153	48/18493
GO:0045061	thymic T cell selection	6.62E-04	**4.97E-03**	17.27	3/153	21/18493
GO:0050857	positive regulation of antigen receptor-mediated signaling pathway	6.62E-04	**4.97E-03**	17.27	3/153	21/18493
GO:0070233	negative regulation of T cell apoptotic process	6.62E-04	**4.97E-03**	17.27	3/153	21/18493
GO:0070542	response to fatty acid	6.63E-04	**4.97E-03**	7.19	5/153	84/18493
GO:0030041	actin filament polymerization	7.09E-04	**5.30E-03**	4.75	7/153	178/18493
GO:0002831	regulation of response to biotic stimulus	7.46E-04	**5.56E-03**	5.58	6/153	130/18493
GO:0070231	T cell apoptotic process	7.72E-04	**5.73E-03**	9.67	4/153	50/18493
GO:0002705	positive regulation of leukocyte mediated immunity	7.77E-04	**5.75E-03**	5.54	6/153	131/18493
GO:0043123	positive regulation of I-kappaB kinase/NF-kappaB signaling	7.82E-04	**5.78E-03**	4.67	7/153	181/18493
GO:0002886	regulation of myeloid leukocyte mediated immunity	8.32E-04	**6.13E-03**	9.48	4/153	51/18493

GO:0050729	positive regulation of inflammatory response	8.41E-04	**6.17E-03**	5.45	6/153	133/18493
GO:0033273	response to vitamin	8.62E-04	**6.26E-03**	6.79	5/153	89/18493
GO:0060337	type I interferon signaling pathway	8.62E-04	**6.26E-03**	6.79	5/153	89/18493
GO:0071357	cellular response to type I interferon	8.62E-04	**6.26E-03**	6.79	5/153	89/18493
GO:0002691	regulation of cellular extravasation	8.71E-04	**6.26E-03**	15.77	3/153	23/18493
GO:0032753	positive regulation of interleukin-4 production	8.71E-04	**6.26E-03**	15.77	3/153	23/18493
GO:0034695	response to prostaglandin E	8.71E-04	**6.26E-03**	15.77	3/153	23/18493
GO:1903020	positive regulation of glycoprotein metabolic process	8.71E-04	**6.26E-03**	15.77	3/153	23/18493
GO:0002763	positive regulation of myeloid leukocyte differentiation	8.96E-04	**6.42E-03**	9.30	4/153	52/18493
GO:0014909	smooth muscle cell migration	9.07E-04	**6.46E-03**	6.71	5/153	90/18493
GO:0045069	regulation of viral genome replication	9.07E-04	**6.46E-03**	6.71	5/153	90/18493
GO:0070588	calcium ion transmembrane transport	9.39E-04	**6.67E-03**	3.60	9/153	302/18493
GO:0002702	positive regulation of production of molecular mediator of immune response	9.53E-04	**6.69E-03**	6.64	5/153	91/18493
GO:0045639	positive regulation of myeloid cell differentiation	9.53E-04	**6.69E-03**	6.64	5/153	91/18493
GO:0046634	regulation of alpha-beta T cell activation	9.53E-04	**6.69E-03**	6.64	5/153	91/18493
GO:2000117	negative regulation of cysteine-type endopeptidase activity	9.53E-04	**6.69E-03**	6.64	5/153	91/18493
GO:0042113	B cell activation	9.61E-04	**6.72E-03**	3.59	9/153	303/18493
GO:0045661	regulation of myoblast differentiation	9.63E-04	**6.72E-03**	9.12	4/153	53/18493
GO:0032535	regulation of cellular component size	9.77E-04	**6.80E-03**	3.29	10/153	367/18493
GO:0060143	positive regulation of syncytium formation by plasma membrane fusion	9.89E-04	**6.87E-03**	15.11	3/153	24/18493
GO:0050730	regulation of peptidyl-tyrosine phosphorylation	1.01E-03	**6.97E-03**	3.95	8/153	245/18493
GO:0050714	positive regulation of protein secretion	1.03E-03	**7.13E-03**	3.93	8/153	246/18493
GO:0045637	regulation of myeloid cell differentiation	1.09E-03	**7.49E-03**	3.90	8/153	248/18493
GO:0034340	response to type I interferon	1.10E-03	**7.57E-03**	6.43	5/153	94/18493
GO:0002230	positive regulation of defense response to virus by host	1.12E-03	**7.62E-03**	14.50	3/153	25/18493
GO:0150077	regulation of neuroinflammatory response	1.12E-03	**7.62E-03**	14.50	3/153	25/18493
GO:0010951	negative regulation of endopeptidase activity	1.15E-03	**7.80E-03**	3.87	8/153	250/18493

GO:0010959	regulation of metal ion transport	1.17E-03	**7.93E-03**	3.21	10/153	376/18493
GO:0032970	regulation of actin filament-based process	1.17E-03	**7.93E-03**	3.21	10/153	376/18493
GO:0001960	negative regulation of cytokine-mediated signaling pathway	1.18E-03	**7.99E-03**	8.63	4/153	56/18493
GO:0002218	activation of innate immune response	1.21E-03	**8.11E-03**	3.84	8/153	252/18493
GO:2001243	negative regulation of intrinsic apoptotic signaling pathway	1.21E-03	**8.13E-03**	6.30	5/153	96/18493
GO:0031954	positive regulation of protein autophosphorylation	1.26E-03	**8.35E-03**	13.95	3/153	26/18493
GO:0043950	positive regulation of cAMP-mediated signaling	1.26E-03	**8.35E-03**	13.95	3/153	26/18493
GO:0051125	regulation of actin nucleation	1.26E-03	**8.35E-03**	13.95	3/153	26/18493
GO:0042108	positive regulation of cytokine biosynthetic process	1.27E-03	**8.40E-03**	8.48	4/153	57/18493
GO:0007187	G protein-coupled receptor signaling pathway, coupled to cyclic nucleotide second messenger	1.33E-03	**8.82E-03**	3.78	8/153	256/18493
GO:0051235	maintenance of location	1.35E-03	**8.88E-03**	3.42	9/153	318/18493
GO:0051092	positive regulation of NF-kappaB transcription factor activity	1.36E-03	**8.96E-03**	4.97	6/153	146/18493
GO:0007229	integrin-mediated signaling pathway	1.39E-03	**9.13E-03**	6.10	5/153	99/18493
GO:0032673	regulation of interleukin-4 production	1.40E-03	**9.19E-03**	13.43	3/153	27/18493
GO:0007259	JAK-STAT cascade	1.41E-03	**9.21E-03**	4.93	6/153	147/18493
GO:0034113	heterotypic cell-cell adhesion	1.44E-03	**9.37E-03**	8.19	4/153	59/18493
GO:0071774	response to fibroblast growth factor	1.46E-03	**9.48E-03**	4.90	6/153	148/18493
GO:0010466	negative regulation of peptidase activity	1.51E-03	**9.75E-03**	3.70	8/153	261/18493
GO:0002286	T cell activation involved in immune response	1.52E-03	**9.78E-03**	5.98	5/153	101/18493
GO:0046632	alpha-beta T cell differentiation	1.52E-03	**9.78E-03**	5.98	5/153	101/18493
GO:0051346	negative regulation of hydrolase activity	1.52E-03	**9.78E-03**	2.90	11/153	458/18493
GO:0010830	regulation of myotube differentiation	1.53E-03	**9.80E-03**	8.06	4/153	60/18493
GO:0060761	negative regulation of response to cytokine stimulus	1.53E-03	**9.80E-03**	8.06	4/153	60/18493
GO:0043304	regulation of mast cell degranulation	1.56E-03	**9.96E-03**	12.95	3/153	28/18493
GO:2001233	regulation of apoptotic signaling pathway	1.57E-03	**9.97E-03**	3.09	10/153	391/18493
GO:0002456	T cell mediated immunity	1.59E-03	**1.00E-02**	5.92	5/153	102/18493

GO:1901222	regulation of NIK/NF-kappaB signaling	1.59E-03	**1.00E-02**	5.92	5/153	102/18493
GO:0046635	positive regulation of alpha-beta T cell activation	1.63E-03	**1.03E-02**	7.93	4/153	61/18493
GO:0014812	muscle cell migration	1.66E-03	**1.04E-02**	5.87	5/153	103/18493
GO:0002793	positive regulation of peptide secretion	1.66E-03	**1.04E-02**	3.65	8/153	265/18493
GO:0030168	platelet activation	1.67E-03	**1.05E-02**	4.77	6/153	152/18493
GO:0032102	negative regulation of response to external stimulus	1.70E-03	**1.06E-02**	3.31	9/153	329/18493
GO:0032623	interleukin-2 production	1.73E-03	**1.07E-02**	7.80	4/153	62/18493
GO:0033006	regulation of mast cell activation involved in immune response	1.73E-03	**1.07E-02**	12.50	3/153	29/18493
GO:0033032	regulation of myeloid cell apoptotic process	1.73E-03	**1.07E-02**	12.50	3/153	29/18493
GO:0046640	regulation of alpha-beta T cell proliferation	1.73E-03	**1.07E-02**	12.50	3/153	29/18493
GO:0050718	positive regulation of interleukin-1 beta secretion	1.73E-03	**1.07E-02**	12.50	3/153	29/18493
GO:0071295	cellular response to vitamin	1.73E-03	**1.07E-02**	12.50	3/153	29/18493
GO:0009266	response to temperature stimulus	1.75E-03	**1.07E-02**	4.07	7/153	208/18493
GO:0010810	regulation of cell-substrate adhesion	1.80E-03	**1.10E-02**	4.05	7/153	209/18493
GO:0002708	positive regulation of lymphocyte mediated immunity	1.80E-03	**1.10E-02**	5.76	5/153	105/18493
GO:0002532	production of molecular mediator involved in inflammatory response	1.84E-03	**1.12E-02**	7.67	4/153	63/18493
GO:0050868	negative regulation of T cell activation	1.88E-03	**1.14E-02**	5.70	5/153	106/18493
GO:0032733	positive regulation of interleukin-10 production	1.91E-03	**1.15E-02**	12.09	3/153	30/18493
GO:0043372	positive regulation of CD4-positive, alpha-beta T cell differentiation	1.91E-03	**1.15E-02**	12.09	3/153	30/18493
GO:0046596	regulation of viral entry into host cell	1.91E-03	**1.15E-02**	12.09	3/153	30/18493
GO:0002260	lymphocyte homeostasis	1.95E-03	**1.17E-02**	7.55	4/153	64/18493
GO:0031343	positive regulation of cell killing	1.95E-03	**1.17E-02**	7.55	4/153	64/18493
GO:0030833	regulation of actin filament polymerization	2.03E-03	**1.22E-02**	4.59	6/153	158/18493
GO:0014902	myotube differentiation	2.04E-03	**1.22E-02**	5.60	5/153	108/18493
GO:0032481	positive regulation of type I interferon production	2.06E-03	**1.22E-02**	7.44	4/153	65/18493
GO:0033627	cell adhesion mediated by integrin	2.06E-03	**1.22E-02**	7.44	4/153	65/18493
GO:0051258	protein polymerization	2.09E-03	**1.24E-02**	3.52	8/153	275/18493

GO:0042554	superoxide anion generation	2.11E-03	**1.24E-02**	11.70	3/153	31/18493
GO:0002292	T cell differentiation involved in immune response	2.18E-03	**1.28E-02**	7.33	4/153	66/18493
GO:0050918	positive chemotaxis	2.18E-03	**1.28E-02**	7.33	4/153	66/18493
GO:2001242	regulation of intrinsic apoptotic signaling pathway	2.24E-03	**1.31E-02**	4.50	6/153	161/18493
GO:0019722	calcium-mediated signaling	2.40E-03	**1.40E-02**	3.85	7/153	220/18493
GO:0031670	cellular response to nutrient	2.43E-03	**1.42E-02**	7.11	4/153	68/18493
GO:0001774	microglial cell activation	2.53E-03	**1.46E-02**	10.99	3/153	33/18493
GO:0002269	leukocyte activation involved in inflammatory response	2.53E-03	**1.46E-02**	10.99	3/153	33/18493
GO:0032633	interleukin-4 production	2.53E-03	**1.46E-02**	10.99	3/153	33/18493
GO:0033028	myeloid cell apoptotic process	2.53E-03	**1.46E-02**	10.99	3/153	33/18493
GO:0032945	negative regulation of mononuclear cell proliferation	2.70E-03	**1.55E-02**	6.91	4/153	70/18493
GO:0050672	negative regulation of lymphocyte proliferation	2.70E-03	**1.55E-02**	6.91	4/153	70/18493
GO:1901224	positive regulation of NIK/NF-kappaB signaling	2.70E-03	**1.55E-02**	6.91	4/153	70/18493
GO:0050716	positive regulation of interleukin-1 secretion	2.75E-03	**1.57E-02**	10.66	3/153	34/18493
GO:2000249	regulation of actin cytoskeleton reorganization	2.75E-03	**1.57E-02**	10.66	3/153	34/18493
GO:2000516	positive regulation of CD4-positive, alpha-beta T cell activation	2.75E-03	**1.57E-02**	10.66	3/153	34/18493
GO:0048660	regulation of smooth muscle cell proliferation	2.77E-03	**1.57E-02**	4.32	6/153	168/18493
GO:0051051	negative regulation of transport	2.79E-03	**1.58E-02**	2.69	11/153	495/18493
GO:0001768	establishment of T cell polarity	2.93E-03	**1.65E-02**	24.17	2/153	10/18493
GO:0071104	response to interleukin-9	2.93E-03	**1.65E-02**	24.17	2/153	10/18493
GO:0048659	smooth muscle cell proliferation	2.93E-03	**1.65E-02**	4.27	6/153	170/18493
GO:0033280	response to vitamin D	2.99E-03	**1.68E-02**	10.36	3/153	35/18493
GO:0043368	positive T cell selection	2.99E-03	**1.68E-02**	10.36	3/153	35/18493
GO:0097305	response to alcohol	3.15E-03	**1.76E-02**	3.66	7/153	231/18493
GO:0038061	NIK/NF-kappaB signaling	3.23E-03	**1.80E-02**	5.04	5/153	120/18493
GO:0043367	CD4-positive, alpha-beta T cell differentiation	3.31E-03	**1.84E-02**	6.53	4/153	74/18493
GO:0046718	viral entry into host cell	3.34E-03	**1.86E-02**	4.99	5/153	121/18493
GO:0050853	B cell receptor signaling pathway	3.46E-03	**1.92E-02**	4.95	5/153	122/18493
GO:0032642	regulation of chemokine production	3.47E-03	**1.92E-02**	6.45	4/153	75/18493

GO:0010831	positive regulation of myotube differentiation	3.51E-03	**1.93E-02**	9.80	3/153	37/18493
GO:0038083	peptidyl-tyrosine autophosphorylation	3.51E-03	**1.93E-02**	9.80	3/153	37/18493
GO:0050856	regulation of T cell receptor signaling pathway	3.51E-03	**1.93E-02**	9.80	3/153	37/18493
GO:0001767	establishment of lymphocyte polarity	3.56E-03	**1.93E-02**	21.98	2/153	11/18493
GO:0033089	positive regulation of T cell differentiation in thymus	3.56E-03	**1.93E-02**	21.98	2/153	11/18493
GO:0033632	regulation of cell-cell adhesion mediated by integrin	3.56E-03	**1.93E-02**	21.98	2/153	11/18493
GO:0045060	negative thymic T cell selection	3.56E-03	**1.93E-02**	21.98	2/153	11/18493
GO:0045953	negative regulation of natural killer cell mediated cytotoxicity	3.56E-03	**1.93E-02**	21.98	2/153	11/18493
GO:0072567	chemokine (C-X-C motif) ligand 2 production	3.56E-03	**1.93E-02**	21.98	2/153	11/18493
GO:1904995	negative regulation of leukocyte adhesion to vascular endothelial cell	3.56E-03	**1.93E-02**	21.98	2/153	11/18493
GO:0010559	regulation of glycoprotein biosynthetic process	3.79E-03	**2.04E-02**	9.54	3/153	38/18493
GO:0019835	cytolysis	4.08E-03	**2.19E-02**	9.30	3/153	39/18493
GO:0033003	regulation of mast cell activation	4.08E-03	**2.19E-02**	9.30	3/153	39/18493
GO:0043029	T cell homeostasis	4.08E-03	**2.19E-02**	9.30	3/153	39/18493
GO:0050864	regulation of B cell activation	4.21E-03	**2.25E-02**	3.96	6/153	183/18493
GO:0002551	mast cell chemotaxis	4.25E-03	**2.25E-02**	20.14	2/153	12/18493
GO:0002716	negative regulation of natural killer cell mediated immunity	4.25E-03	**2.25E-02**	20.14	2/153	12/18493
GO:0038110	interleukin-2-mediated signaling pathway	4.25E-03	**2.25E-02**	20.14	2/153	12/18493
GO:0043383	negative T cell selection	4.25E-03	**2.25E-02**	20.14	2/153	12/18493
GO:0043922	negative regulation by host of viral transcription	4.25E-03	**2.25E-02**	20.14	2/153	12/18493
GO:0051279	regulation of release of sequestered calcium ion into cytosol	4.38E-03	**2.29E-02**	6.04	4/153	80/18493
GO:0002820	negative regulation of adaptive immune response	4.38E-03	**2.29E-02**	9.07	3/153	40/18493
GO:0030890	positive regulation of B cell proliferation	4.38E-03	**2.29E-02**	9.07	3/153	40/18493
GO:0051281	positive regulation of release of sequestered calcium ion into cytosol	4.38E-03	**2.29E-02**	9.07	3/153	40/18493
GO:0061900	glial cell activation	4.38E-03	**2.29E-02**	9.07	3/153	40/18493
GO:0045765	regulation of angiogenesis	4.51E-03	**2.35E-02**	2.86	9/153	381/18493

GO:0050731	positive regulation of peptidyl-tyrosine phosphorylation	4.55E-03	**2.37E-02**	3.90	6/153	186/18493
GO:0030574	collagen catabolic process	4.70E-03	**2.44E-02**	8.84	3/153	41/18493
GO:0032602	chemokine production	4.78E-03	**2.48E-02**	5.90	4/153	82/18493
GO:0071352	cellular response to interleukin-2	5.00E-03	**2.54E-02**	18.60	2/153	13/18493
GO:0097531	mast cell migration	5.00E-03	**2.54E-02**	18.60	2/153	13/18493
GO:1900025	negative regulation of substrate adhesion-dependent cell spreading	5.00E-03	**2.54E-02**	18.60	2/153	13/18493
GO:0002700	regulation of production of molecular mediator of immune response	5.00E-03	**2.54E-02**	4.54	5/153	133/18493
GO:0030260	entry into host cell	5.00E-03	**2.54E-02**	4.54	5/153	133/18493
GO:0044409	entry into host	5.00E-03	**2.54E-02**	4.54	5/153	133/18493
GO:0051806	entry into cell of other organism involved in symbiotic interaction	5.00E-03	**2.54E-02**	4.54	5/153	133/18493
GO:0051828	entry into other organism involved in symbiotic interaction	5.00E-03	**2.54E-02**	4.54	5/153	133/18493
GO:1904892	regulation of STAT cascade	5.00E-03	**2.54E-02**	4.54	5/153	133/18493
GO:0045429	positive regulation of nitric oxide biosynthetic process	5.03E-03	**2.55E-02**	8.63	3/153	42/18493
GO:0050706	regulation of interleukin-1 beta secretion	5.03E-03	**2.55E-02**	8.63	3/153	42/18493
GO:1904407	positive regulation of nitric oxide metabolic process	5.03E-03	**2.55E-02**	8.63	3/153	42/18493
GO:0043491	protein kinase B signaling	5.16E-03	**2.61E-02**	3.34	7/153	253/18493
GO:0007565	female pregnancy	5.17E-03	**2.61E-02**	3.80	6/153	191/18493
GO:0043154	negative regulation of cysteine-type endopeptidase activity involved in apoptotic process	5.21E-03	**2.61E-02**	5.76	4/153	84/18493
GO:0097306	cellular response to alcohol	5.21E-03	**2.61E-02**	5.76	4/153	84/18493
GO:0002792	negative regulation of peptide secretion	5.32E-03	**2.66E-02**	4.48	5/153	135/18493
GO:0051091	positive regulation of DNA-binding transcription factor activity	5.39E-03	**2.69E-02**	3.32	7/153	255/18493
GO:0045445	myoblast differentiation	5.43E-03	**2.71E-02**	5.69	4/153	85/18493
GO:0002639	positive regulation of immunoglobulin production	5.74E-03	**2.84E-02**	8.24	3/153	44/18493
GO:0033628	regulation of cell adhesion mediated by integrin	5.74E-03	**2.84E-02**	8.24	3/153	44/18493
GO:0032823	regulation of natural killer cell differentiation	5.80E-03	**2.84E-02**	17.27	2/153	14/18493

GO:0051770	positive regulation of nitric-oxide synthase biosynthetic process	5.80E-03	**2.84E-02**	17.27	2/153	14/18493
GO:0060263	regulation of respiratory burst	5.80E-03	**2.84E-02**	17.27	2/153	14/18493
GO:0070669	response to interleukin-2	5.80E-03	**2.84E-02**	17.27	2/153	14/18493
GO:0071361	cellular response to ethanol	5.80E-03	**2.84E-02**	17.27	2/153	14/18493
GO:2000402	negative regulation of lymphocyte migration	5.80E-03	**2.84E-02**	17.27	2/153	14/18493
GO:0048871	multicellular organismal homeostasis	5.89E-03	**2.88E-02**	2.57	10/153	471/18493
GO:0035710	CD4-positive, alpha-beta T cell activation	5.89E-03	**2.88E-02**	5.56	4/153	87/18493
GO:0007189	adenylate cyclase-activating G protein-coupled receptor signaling pathway	6.01E-03	**2.93E-02**	4.35	5/153	139/18493
GO:1903018	regulation of glycoprotein metabolic process	6.11E-03	**2.97E-02**	8.06	3/153	45/18493
GO:1904894	positive regulation of STAT cascade	6.13E-03	**2.98E-02**	5.49	4/153	88/18493
GO:0043062	extracellular structure organization	6.35E-03	**3.07E-02**	2.71	9/153	402/18493
GO:0045807	positive regulation of endocytosis	6.38E-03	**3.08E-02**	4.29	5/153	141/18493
GO:0044344	cellular response to fibroblast growth factor stimulus	6.57E-03	**3.16E-02**	4.26	5/153	142/18493
GO:0106106	cold-induced thermogenesis	6.57E-03	**3.16E-02**	4.26	5/153	142/18493
GO:0120161	regulation of cold-induced thermogenesis	6.57E-03	**3.16E-02**	4.26	5/153	142/18493
GO:0002830	positive regulation of type 2 immune response	6.65E-03	**3.16E-02**	16.12	2/153	15/18493
GO:0030852	regulation of granulocyte differentiation	6.65E-03	**3.16E-02**	16.12	2/153	15/18493
GO:0033631	cell-cell adhesion mediated by integrin	6.65E-03	**3.16E-02**	16.12	2/153	15/18493
GO:0050961	detection of temperature stimulus involved in sensory perception	6.65E-03	**3.16E-02**	16.12	2/153	15/18493
GO:0050965	detection of temperature stimulus involved in sensory perception of pain	6.65E-03	**3.16E-02**	16.12	2/153	15/18493
GO:2000484	positive regulation of interleukin-8 secretion	6.65E-03	**3.16E-02**	16.12	2/153	15/18493
GO:0031667	response to nutrient levels	6.79E-03	**3.22E-02**	2.51	10/153	481/18493
GO:0002720	positive regulation of cytokine production involved in immune response	6.90E-03	**3.25E-02**	7.72	3/153	47/18493
GO:0043370	regulation of CD4-positive, alpha-beta T cell differentiation	6.90E-03	**3.25E-02**	7.72	3/153	47/18493
GO:0046638	positive regulation of alpha-beta T cell differentiation	6.90E-03	**3.25E-02**	7.72	3/153	47/18493

GO:0002822	regulation of adaptive immune response based on somatic recombination of immune receptors built from immunoglobulin superfamily domains	6.96E-03	**3.27E-02**	4.20	5/153	144/18493
GO:0002440	production of molecular mediator of immune response	7.01E-03	**3.29E-02**	3.16	7/153	268/18493
GO:1903169	regulation of calcium ion transmembrane transport	7.16E-03	**3.35E-02**	4.17	5/153	145/18493
GO:0050702	interleukin-1 beta secretion	7.32E-03	**3.41E-02**	7.55	3/153	48/18493
GO:0098586	cellular response to virus	7.32E-03	**3.41E-02**	7.55	3/153	48/18493
GO:0032930	positive regulation of superoxide anion generation	7.56E-03	**3.51E-02**	15.11	2/153	16/18493
GO:0033033	negative regulation of myeloid cell apoptotic process	7.56E-03	**3.51E-02**	15.11	2/153	16/18493
GO:2001267	regulation of cysteine-type endopeptidase activity involved in apoptotic signaling pathway	7.56E-03	**3.51E-02**	15.11	2/153	16/18493
GO:0006909	phagocytosis	7.70E-03	**3.56E-02**	2.83	8/153	342/18493
GO:0042035	regulation of cytokine biosynthetic process	7.72E-03	**3.56E-02**	5.14	4/153	94/18493
GO:0046456	icosanoid biosynthetic process	7.75E-03	**3.56E-02**	7.40	3/153	49/18493
GO:0050704	regulation of interleukin-1 secretion	7.75E-03	**3.56E-02**	7.40	3/153	49/18493
GO:0032271	regulation of protein polymerization	7.75E-03	**3.56E-02**	3.49	6/153	208/18493
GO:1903305	regulation of regulated secretory pathway	7.79E-03	**3.57E-02**	4.08	5/153	148/18493
GO:0043281	regulation of cysteine-type endopeptidase activity involved in apoptotic process	7.93E-03	**3.62E-02**	3.47	6/153	209/18493
GO:0051701	interaction with host	7.93E-03	**3.62E-02**	3.47	6/153	209/18493
GO:0043434	response to peptide hormone	7.98E-03	**3.64E-02**	2.61	9/153	417/18493
GO:0030316	osteoclast differentiation	8.01E-03	**3.65E-02**	5.09	4/153	95/18493
GO:0001913	T cell mediated cytotoxicity	8.19E-03	**3.71E-02**	7.25	3/153	50/18493
GO:0045010	actin nucleation	8.19E-03	**3.71E-02**	7.25	3/153	50/18493
GO:0006898	receptor-mediated endocytosis	8.23E-03	**3.72E-02**	2.79	8/153	346/18493
GO:1990845	adaptive thermogenesis	8.23E-03	**3.72E-02**	4.03	5/153	150/18493
GO:0031532	actin cytoskeleton reorganization	8.31E-03	**3.74E-02**	5.04	4/153	96/18493
GO:0120162	positive regulation of cold-induced thermogenesis	8.31E-03	**3.74E-02**	5.04	4/153	96/18493
GO:0090066	regulation of anatomical structure size	8.46E-03	**3.75E-02**	2.43	10/153	497/18493
GO:1901342	regulation of vasculature development	8.47E-03	**3.75E-02**	2.58	9/153	421/18493

GO:0016064	immunoglobulin mediated immune response	8.48E-03	**3.75E-02**	3.42	6/153	212/18493
GO:0030198	extracellular matrix organization	8.50E-03	**3.75E-02**	2.78	8/153	348/18493
GO:0002418	immune response to tumor cell	8.52E-03	**3.75E-02**	14.22	2/153	17/18493
GO:0002689	negative regulation of leukocyte chemotaxis	8.52E-03	**3.75E-02**	14.22	2/153	17/18493
GO:0002710	negative regulation of T cell mediated immunity	8.52E-03	**3.75E-02**	14.22	2/153	17/18493
GO:0061081	positive regulation of myeloid leukocyte cytokine production involved in immune response	8.52E-03	**3.75E-02**	14.22	2/153	17/18493
GO:0071605	monocyte chemotactic protein-1 production	8.52E-03	**3.75E-02**	14.22	2/153	17/18493
GO:0071637	regulation of monocyte chemotactic protein-1 production	8.52E-03	**3.75E-02**	14.22	2/153	17/18493
GO:0090280	positive regulation of calcium ion import	8.52E-03	**3.75E-02**	14.22	2/153	17/18493
GO:1903978	regulation of microglial cell activation	8.52E-03	**3.75E-02**	14.22	2/153	17/18493
GO:0060627	regulation of vesicle-mediated transport	8.57E-03	**3.77E-02**	2.427093997	10/153	498/18493
GO:0007254	JNK cascade	8.66E-03	**3.79E-02**	3.40476848	6/153	213/18493
GO:0019724	B cell mediated immunity	8.66E-03	**3.79E-02**	3.40	6/153	213/18493
GO:0030838	positive regulation of actin filament polymerization	8.92E-03	**3.89E-02**	4.93	4/153	98/18493
GO:0048661	positive regulation of smooth muscle cell proliferation	8.92E-03	**3.89E-02**	4.93	4/153	98/18493
GO:2000379	positive regulation of reactive oxygen species metabolic process	8.92E-03	**3.89E-02**	4.93	4/153	98/18493
GO:0032720	negative regulation of tumor necrosis factor production	9.13E-03	**3.96E-02**	6.97	3/153	52/18493
GO:0043030	regulation of macrophage activation	9.13E-03	**3.96E-02**	6.97	3/153	52/18493
GO:0051403	stress-activated MAPK cascade	9.49E-03	**4.11E-02**	2.98	7/153	284/18493
GO:0032816	positive regulation of natural killer cell activation	9.54E-03	**4.12E-02**	13.43	2/153	18/18493
GO:0002824	positive regulation of adaptive immune response based on somatic recombination of immune receptors built from immunoglobulin superfamily domains	9.56E-03	**4.12E-02**	4.83	4/153	100/18493
GO:0010522	regulation of calcium ion transport into cytosol	9.56E-03	**4.12E-02**	4.83	4/153	100/18493
GO:0031529	ruffle organization	9.62E-03	**4.12E-02**	6.84	3/153	53/18493
GO:0032663	regulation of interleukin-2 production	9.62E-03	**4.12E-02**	6.84	3/153	53/18493
GO:0032722	positive regulation of chemokine production	9.62E-03	**4.12E-02**	6.84	3/153	53/18493
GO:0097193	intrinsic apoptotic signaling pathway	1.00E-02	**4.28E-02**	2.95	7/153	287/18493

GO:0051495	positive regulation of cytoskeleton organization	1.01E-02	**4.30E-02**	3.30	6/153	220/18493
GO:0010524	positive regulation of calcium ion transport into cytosol	1.01E-02	**4.30E-02**	6.71	3/153	54/18493
GO:0043949	regulation of cAMP-mediated signaling	1.01E-02	**4.30E-02**	6.71	3/153	54/18493
GO:1903556	negative regulation of tumor necrosis factor superfamily cytokine production	1.01E-02	**4.30E-02**	6.71	3/153	54/18493
GO:0045861	negative regulation of proteolysis	1.01E-02	**4.30E-02**	2.69	8/153	359/18493
GO:0008630	intrinsic apoptotic signaling pathway in response to DNA damage	1.02E-02	**4.33E-02**	4.74	4/153	102/18493
GO:0007188	adenylate cyclase-modulating G protein-coupled receptor signaling pathway	1.03E-02	**4.34E-02**	3.28	6/153	221/18493
GO:0044706	multi-multicellular organism process	1.03E-02	**4.34E-02**	3.28	6/153	221/18493
GO:0007160	cell-matrix adhesion	1.05E-02	**4.40E-02**	3.27	6/153	222/18493
GO:0051048	negative regulation of secretion	1.05E-02	**4.40E-02**	3.27	6/153	222/18493
GO:2001234	negative regulation of apoptotic signaling pathway	1.05E-02	**4.40E-02**	3.27	6/153	222/18493
GO:0010829	negative regulation of glucose transmembrane transport	1.06E-02	**4.41E-02**	12.72	2/153	19/18493
GO:0016048	detection of temperature stimulus	1.06E-02	**4.41E-02**	12.72	2/153	19/18493
GO:0030502	negative regulation of bone mineralization	1.06E-02	**4.41E-02**	12.72	2/153	19/18493
GO:0046641	positive regulation of alpha-beta T cell proliferation	1.06E-02	**4.41E-02**	12.72	2/153	19/18493
GO:0050860	negative regulation of T cell receptor signaling pathway	1.06E-02	**4.41E-02**	12.72	2/153	19/18493
GO:0090382	phagosome maturation	1.06E-02	**4.41E-02**	12.72	2/153	19/18493
GO:0001912	positive regulation of leukocyte mediated cytotoxicity	1.06E-02	**4.41E-02**	6.59	3/153	55/18493
GO:1903428	positive regulation of reactive oxygen species biosynthetic process	1.06E-02	**4.41E-02**	6.59	3/153	55/18493
GO:0042130	negative regulation of T cell proliferation	1.12E-02	**4.61E-02**	6.48	3/153	56/18493
GO:0050701	interleukin-1 secretion	1.12E-02	**4.61E-02**	6.48	3/153	56/18493
GO:0002821	positive regulation of adaptive immune response	1.13E-02	**4.64E-02**	4.60	4/153	105/18493
GO:0042089	cytokine biosynthetic process	1.13E-02	**4.64E-02**	4.60	4/153	105/18493
GO:0046777	protein autophosphorylation	1.16E-02	**4.76E-02**	3.19	6/153	227/18493
GO:0042107	cytokine metabolic process	1.17E-02	**4.76E-02**	4.56	4/153	106/18493
GO:0031579	membrane raft organization	1.17E-02	**4.76E-02**	12.09	2/153	20/18493

GO:0032682	negative regulation of chemokine production	1.17E-02	**4.76E-02**	12.09	2/153	20/18493
GO:0032928	regulation of superoxide anion generation	1.17E-02	**4.76E-02**	12.09	2/153	20/18493
GO:0071305	cellular response to vitamin D	1.17E-02	**4.76E-02**	12.09	2/153	20/18493
GO:0050766	positive regulation of phagocytosis	1.17E-02	**4.76E-02**	6.36	3/153	57/18493
GO:2000514	regulation of CD4-positive, alpha-beta T cell activation	1.17E-02	**4.76E-02**	6.36	3/153	57/18493
GO:0031669	cellular response to nutrient levels	1.18E-02	**4.80E-02**	3.18	6/153	228/18493
GO:0002294	CD4-positive, alpha-beta T cell differentiation involved in immune response	1.29E-02	**5.17E-02**	6.15	3/153	59/18493
GO:0019883	antigen processing and presentation of endogenous antigen	1.29E-02	**5.17E-02**	11.51	2/153	21/18493
GO:0043302	positive regulation of leukocyte degranulation	1.29E-02	**5.17E-02**	11.51	2/153	21/18493
GO:0050951	sensory perception of temperature stimulus	1.29E-02	**5.17E-02**	11.51	2/153	21/18493
GO:1904994	regulation of leukocyte adhesion to vascular endothelial cell	1.29E-02	**5.17E-02**	11.51	2/153	21/18493
GO:0051897	positive regulation of protein kinase B signaling	1.30E-02	**5.21E-02**	3.60	5/153	168/18493
GO:0002758	innate immune response-activating signal transduction	1.31E-02	**5.23E-02**	3.11	6/153	233/18493
GO:0002287	alpha-beta T cell activation involved in immune response	1.35E-02	**5.37E-02**	6.04	3/153	60/18493
GO:0002293	alpha-beta T cell differentiation involved in immune response	1.35E-02	**5.37E-02**	6.04	3/153	60/18493
GO:1903902	positive regulation of viral life cycle	1.35E-02	**5.37E-02**	6.04	3/153	60/18493
GO:0048771	tissue remodeling	1.36E-02	**5.40E-02**	3.55	5/153	170/18493
GO:2001235	positive regulation of apoptotic signaling pathway	1.36E-02	**5.40E-02**	3.55	5/153	170/18493
GO:0002698	negative regulation of immune effector process	1.36E-02	**5.40E-02**	4.36	4/153	111/18493
GO:0033002	muscle cell proliferation	1.38E-02	**5.48E-02**	3.07	6/153	236/18493
GO:0001503	ossification	1.39E-02	**5.49E-02**	2.54	8/153	380/18493
GO:0001659	temperature homeostasis	1.39E-02	**5.50E-02**	3.53	5/153	171/18493
GO:0002347	response to tumor cell	1.41E-02	**5.50E-02**	10.99	2/153	22/18493
GO:0006925	inflammatory cell apoptotic process	1.41E-02	**5.50E-02**	10.99	2/153	22/18493
GO:0032703	negative regulation of interleukin-2 production	1.41E-02	**5.50E-02**	10.99	2/153	22/18493
GO:0032727	positive regulation of interferon-alpha production	1.41E-02	**5.50E-02**	10.99	2/153	22/18493

GO:0042454	ribonucleoside catabolic process	1.41E-02	**5.50E-02**	10.99	2/153	22/18493
GO:0045663	positive regulation of myoblast differentiation	1.41E-02	**5.50E-02**	10.99	2/153	22/18493
GO:0071379	cellular response to prostaglandin stimulus	1.41E-02	**5.50E-02**	10.99	2/153	22/18493
GO:0032479	regulation of type I interferon production	1.45E-02	**5.62E-02**	4.28	4/153	113/18493
GO:0051153	regulation of striated muscle cell differentiation	1.45E-02	**5.62E-02**	4.28	4/153	113/18493
GO:0045428	regulation of nitric oxide biosynthetic process	1.47E-02	**5.71E-02**	5.85	3/153	62/18493
GO:0032606	type I interferon production	1.49E-02	**5.77E-02**	4.24	4/153	114/18493
GO:1903409	reactive oxygen species biosynthetic process	1.53E-02	**5.90E-02**	4.20	4/153	115/18493
GO:0010499	proteasomal ubiquitin-independent protein catabolic process	1.54E-02	**5.90E-02**	10.51	2/153	23/18493
GO:0071404	cellular response to low-density lipoprotein particle stimulus	1.54E-02	**5.90E-02**	10.51	2/153	23/18493
GO:0002637	regulation of immunoglobulin production	1.54E-02	**5.90E-02**	5.76	3/153	63/18493
GO:0046637	regulation of alpha-beta T cell differentiation	1.54E-02	**5.90E-02**	5.76	3/153	63/18493
GO:0031098	stress-activated protein kinase signaling cascade	1.55E-02	**5.95E-02**	2.70	7/153	313/18493
GO:1904062	regulation of cation transmembrane transport	1.58E-02	**6.04E-02**	2.69	7/153	314/18493
GO:0045670	regulation of osteoclast differentiation	1.60E-02	**6.12E-02**	5.67	3/153	64/18493
GO:0050710	negative regulation of cytokine secretion	1.60E-02	**6.12E-02**	5.67	3/153	64/18493
GO:0001952	regulation of cell-matrix adhesion	1.62E-02	**6.20E-02**	4.13	4/153	117/18493
GO:0001516	prostaglandin biosynthetic process	1.67E-02	**6.29E-02**	10.07	2/153	24/18493
GO:0032897	negative regulation of viral transcription	1.67E-02	**6.29E-02**	10.07	2/153	24/18493
GO:0033081	regulation of T cell differentiation in thymus	1.67E-02	**6.29E-02**	10.07	2/153	24/18493
GO:0046457	prostanoid biosynthetic process	1.67E-02	**6.29E-02**	10.07	2/153	24/18493
GO:0050855	regulation of B cell receptor signaling pathway	1.67E-02	**6.29E-02**	10.07	2/153	24/18493
GO:2000482	regulation of interleukin-8 secretion	1.67E-02	**6.29E-02**	10.07	2/153	24/18493
GO:0002377	immunoglobulin production	1.70E-02	**6.43E-02**	3.36	5/153	180/18493
GO:0002709	regulation of T cell mediated immunity	1.74E-02	**6.53E-02**	5.49	3/153	66/18493
GO:0010812	negative regulation of cell-substrate adhesion	1.74E-02	**6.53E-02**	5.49	3/153	66/18493
GO:0032677	regulation of interleukin-8 production	1.74E-02	**6.53E-02**	5.49	3/153	66/18493
GO:0019751	polyol metabolic process	1.77E-02	**6.62E-02**	4.03	4/153	120/18493

GO:0002507	tolerance induction	1.80E-02	**6.71E-02**	9.67	2/153	25/18493
GO:0032461	positive regulation of protein oligomerization	1.80E-02	**6.71E-02**	9.67	2/153	25/18493
GO:0045662	negative regulation of myoblast differentiation	1.80E-02	**6.71E-02**	9.67	2/153	25/18493
GO:0050858	negative regulation of antigen receptor-mediated signaling pathway	1.80E-02	**6.71E-02**	9.67	2/153	25/18493
GO:0071902	positive regulation of protein serine/threonine kinase activity	1.81E-02	**6.74E-02**	2.62	7/153	323/18493
GO:0046328	regulation of JNK cascade	1.86E-02	**6.89E-02**	3.28	5/153	184/18493
GO:1901652	response to peptide	1.88E-02	**6.96E-02**	2.26	9/153	481/18493
GO:2000377	regulation of reactive oxygen species metabolic process	1.89E-02	**7.02E-02**	3.27	5/153	185/18493
GO:0019933	cAMP-mediated signaling	1.93E-02	**7.15E-02**	3.25	5/153	186/18493
GO:0070168	negative regulation of biomineral tissue development	1.94E-02	**7.17E-02**	9.30	2/153	26/18493
GO:0006801	superoxide metabolic process	1.95E-02	**7.21E-02**	5.26	3/153	69/18493
GO:0034763	negative regulation of transmembrane transport	1.97E-02	**7.24E-02**	3.90	4/153	124/18493
GO:0031099	regeneration	2.02E-02	**7.41E-02**	3.21	5/153	188/18493
GO:0042531	positive regulation of tyrosine phosphorylation of STAT protein	2.03E-02	**7.43E-02**	5.18	3/153	70/18493
GO:0048662	negative regulation of smooth muscle cell proliferation	2.03E-02	**7.43E-02**	5.18	3/153	70/18493
GO:1904427	positive regulation of calcium ion transmembrane transport	2.03E-02	**7.43E-02**	5.18	3/153	70/18493
GO:0043406	positive regulation of MAP kinase activity	2.05E-02	**7.51E-02**	2.81	6/153	258/18493
GO:0001101	response to acid chemical	2.07E-02	**7.55E-02**	2.55	7/153	332/18493
GO:0002920	regulation of humoral immune response	2.07E-02	**7.55E-02**	3.84	4/153	126/18493
GO:0043921	modulation by host of viral transcription	2.08E-02	**7.56E-02**	8.95	2/153	27/18493
GO:0052472	modulation by host of symbiont transcription	2.08E-02	**7.56E-02**	8.95	2/153	27/18493
GO:1990776	response to angiotensin	2.08E-02	**7.56E-02**	8.95	2/153	27/18493
GO:1901654	response to ketone	2.10E-02	**7.60E-02**	3.18	5/153	190/18493
GO:0051155	positive regulation of striated muscle cell differentiation	2.11E-02	**7.62E-02**	5.11	3/153	71/18493
GO:0045471	response to ethanol	2.13E-02	**7.68E-02**	3.81	4/153	127/18493
GO:0007596	blood coagulation	2.13E-02	**7.68E-02**	2.53	7/153	334/18493

GO:0043405	regulation of MAP kinase activity	2.16E-02	**7.78E-02**	2.53	7/153	335/18493
GO:0050709	negative regulation of protein secretion	2.18E-02	**7.84E-02**	3.78	4/153	128/18493
GO:0009988	cell-cell recognition	2.19E-02	**7.84E-02**	5.04	3/153	72/18493
GO:0030278	regulation of ossification	2.23E-02	**7.93E-02**	3.13	5/153	193/18493
GO:0002888	positive regulation of myeloid leukocyte mediated immunity	2.23E-02	**7.93E-02**	8.63	2/153	28/18493
GO:0032647	regulation of interferon-alpha production	2.23E-02	**7.93E-02**	8.63	2/153	28/18493
GO:0052312	modulation of transcription in other organism involved in symbiotic interaction	2.23E-02	**7.93E-02**	8.63	2/153	28/18493
GO:0061082	myeloid leukocyte cytokine production	2.23E-02	**7.93E-02**	8.63	2/153	28/18493
GO:0072606	interleukin-8 secretion	2.23E-02	**7.93E-02**	8.63	2/153	28/18493
GO:0032273	positive regulation of protein polymerization	2.24E-02	**7.93E-02**	3.75	4/153	129/18493
GO:0046425	regulation of JAK-STAT cascade	2.24E-02	**7.93E-02**	3.75	4/153	129/18493
GO:0006809	nitric oxide biosynthetic process	2.27E-02	**8.01E-02**	4.97	3/153	73/18493
GO:0032637	interleukin-8 production	2.27E-02	**8.01E-02**	4.97	3/153	73/18493
GO:0007599	hemostasis	2.29E-02	**8.08E-02**	2.50	7/153	339/18493
GO:0050817	coagulation	2.32E-02	**8.18E-02**	2.49	7/153	340/18493
GO:0022617	extracellular matrix disassembly	2.35E-02	**8.26E-02**	4.90	3/153	74/18493
GO:1903531	negative regulation of secretion by cell	2.36E-02	**8.30E-02**	3.08	5/153	196/18493
GO:0032607	interferon-alpha production	2.39E-02	**8.35E-02**	8.34	2/153	29/18493
GO:0045954	positive regulation of natural killer cell mediated cytotoxicity	2.39E-02	**8.35E-02**	8.34	2/153	29/18493
GO:0050869	negative regulation of B cell activation	2.39E-02	**8.35E-02**	8.34	2/153	29/18493
GO:0070373	negative regulation of ERK1 and ERK2 cascade	2.43E-02	**8.50E-02**	4.83	3/153	75/18493
GO:0001773	myeloid dendritic cell activation	2.54E-02	**8.80E-02**	8.06	2/153	30/18493
GO:0002828	regulation of type 2 immune response	2.54E-02	**8.80E-02**	8.06	2/153	30/18493
GO:0010543	regulation of platelet activation	2.54E-02	**8.80E-02**	8.06	2/153	30/18493
GO:0032743	positive regulation of interleukin-2 production	2.54E-02	**8.80E-02**	8.06	2/153	30/18493
GO:0032814	regulation of natural killer cell activation	2.54E-02	**8.80E-02**	8.06	2/153	30/18493
GO:0038111	interleukin-7-mediated signaling pathway	2.54E-02	**8.80E-02**	8.06	2/153	30/18493

GO:0042590	antigen processing and presentation of exogenous peptide antigen via MHC class I	2.54E-02	**8.80E-02**	8.06	2/153	30/18493
GO:1902905	positive regulation of supramolecular fiber organization	2.55E-02	**8.81E-02**	3.02	5/153	200/18493
GO:0002718	regulation of cytokine production involved in immune response	2.60E-02	**8.97E-02**	4.71	3/153	77/18493
GO:0046209	nitric oxide metabolic process	2.60E-02	**8.97E-02**	4.71	3/153	77/18493
GO:0014065	phosphatidylinositol 3-kinase signaling	2.65E-02	**9.11E-02**	3.55	4/153	136/18493
GO:1904064	positive regulation of cation transmembrane transport	2.65E-02	**9.11E-02**	3.55	4/153	136/18493
GO:0031100	animal organ regeneration	2.69E-02	**9.16E-02**	4.65	3/153	78/18493
GO:0071260	cellular response to mechanical stimulus	2.69E-02	**9.16E-02**	4.65	3/153	78/18493
GO:0002717	positive regulation of natural killer cell mediated immunity	2.70E-02	**9.16E-02**	7.80	2/153	31/18493
GO:0043552	positive regulation of phosphatidylinositol 3-kinase activity	2.70E-02	**9.16E-02**	7.80	2/153	31/18493
GO:0045589	regulation of regulatory T cell differentiation	2.70E-02	**9.16E-02**	7.80	2/153	31/18493
GO:0048873	homeostasis of number of cells within a tissue	2.70E-02	**9.16E-02**	7.80	2/153	31/18493
GO:0055094	response to lipoprotein particle	2.70E-02	**9.16E-02**	7.80	2/153	31/18493
GO:0098760	response to interleukin-7	2.70E-02	**9.16E-02**	7.80	2/153	31/18493
GO:0098761	cellular response to interleukin-7	2.70E-02	**9.16E-02**	7.80	2/153	31/18493
GO:1902230	negative regulation of intrinsic apoptotic signaling pathway in response to DNA damage	2.70E-02	**9.16E-02**	7.80	2/153	31/18493
GO:0072593	reactive oxygen species metabolic process	2.74E-02	**9.26E-02**	2.63	6/153	276/18493
GO:0017157	regulation of exocytosis	2.75E-02	**9.26E-02**	2.96	5/153	204/18493
GO:0032869	cellular response to insulin stimulus	2.75E-02	**9.26E-02**	2.96	5/153	204/18493
GO:0050728	negative regulation of inflammatory response	2.84E-02	**9.58E-02**	3.48	4/153	139/18493
GO:0030851	granulocyte differentiation	2.87E-02	**9.59E-02**	7.55	2/153	32/18493
GO:0048841	regulation of axon extension involved in axon guidance	2.87E-02	**9.59E-02**	7.55	2/153	32/18493
GO:0071353	cellular response to interleukin-4	2.87E-02	**9.59E-02**	7.55	2/153	32/18493
GO:0042509	regulation of tyrosine phosphorylation of STAT protein	2.87E-02	**9.59E-02**	4.53	3/153	80/18493
GO:0050764	regulation of phagocytosis	2.87E-02	**9.59E-02**	4.53	3/153	80/18493
GO:2001057	reactive nitrogen species metabolic process	2.87E-02	**9.59E-02**	4.53	3/153	80/18493

GO:0046330	positive regulation of JNK cascade	2.91E-02	**9.70E-02**	3.45	4/153	140/18493
GO:0001914	regulation of T cell mediated cytotoxicity	3.04E-02	**1.00E-01**	7.33	2/153	33/18493
GO:0009164	nucleoside catabolic process	3.04E-02	**1.00E-01**	7.33	2/153	33/18493
GO:0016601	Rac protein signal transduction	3.04E-02	**1.00E-01**	7.33	2/153	33/18493
GO:0030947	regulation of vascular endothelial growth factor receptor signaling pathway	3.04E-02	**1.00E-01**	7.33	2/153	33/18493
GO:0045066	regulatory T cell differentiation	3.04E-02	**1.00E-01**	7.33	2/153	33/18493
GO:0071354	cellular response to interleukin-6	3.04E-02	**1.00E-01**	7.33	2/153	33/18493
GO:0071402	cellular response to lipoprotein particle stimulus	3.04E-02	**1.00E-01**	7.33	2/153	33/18493
GO:2001056	positive regulation of cysteine-type endopeptidase activity	3.11E-02	**1.03E-01**	3.38	4/153	143/18493
GO:0002526	acute inflammatory response	3.11E-02	**1.03E-01**	2.86	5/153	211/18493
GO:0007009	plasma membrane organization	3.15E-02	**1.04E-01**	4.37	3/153	83/18493
GO:0007260	tyrosine phosphorylation of STAT protein	3.15E-02	**1.04E-01**	4.37	3/153	83/18493
GO:0010922	positive regulation of phosphatase activity	3.21E-02	**1.05E-01**	7.11	2/153	34/18493
GO:0030224	monocyte differentiation	3.21E-02	**1.05E-01**	7.11	2/153	34/18493
GO:0090322	regulation of superoxide metabolic process	3.21E-02	**1.05E-01**	7.11	2/153	34/18493
GO:1903131	mononuclear cell differentiation	3.21E-02	**1.05E-01**	7.11	2/153	34/18493
GO:0008277	regulation of G protein-coupled receptor signaling pathway	3.25E-02	**1.06E-01**	3.33	4/153	145/18493
GO:0019935	cyclic-nucleotide-mediated signaling	3.28E-02	**1.07E-01**	2.82	5/153	214/18493
GO:0002262	myeloid cell homeostasis	3.32E-02	**1.08E-01**	3.31	4/153	146/18493
GO:0046427	positive regulation of JAK-STAT cascade	3.35E-02	**1.09E-01**	4.27	3/153	85/18493
GO:0048246	macrophage chemotaxis	3.39E-02	**1.10E-01**	6.91	2/153	35/18493
GO:0070670	response to interleukin-4	3.39E-02	**1.10E-01**	6.91	2/153	35/18493
GO:0090218	positive regulation of lipid kinase activity	3.39E-02	**1.10E-01**	6.91	2/153	35/18493
GO:0048524	positive regulation of viral process	3.45E-02	**1.12E-01**	4.22	3/153	86/18493
GO:0034767	positive regulation of ion transmembrane transport	3.46E-02	**1.12E-01**	3.27	4/153	148/18493
GO:0072330	monocarboxylic acid biosynthetic process	3.52E-02	**1.13E-01**	2.48	6/153	293/18493
GO:0034765	regulation of ion transmembrane transport	3.53E-02	**1.13E-01**	2.13	8/153	454/18493

GO:0002455	humoral immune response mediated by circulating immunoglobulin	3.54E-02	**1.13E-01**	3.24	4/153	149/18493
GO:0002673	regulation of acute inflammatory response	3.54E-02	**1.13E-01**	3.24	4/153	149/18493
GO:0061025	membrane fusion	3.54E-02	**1.13E-01**	3.24	4/153	149/18493
GO:0006692	prostanoid metabolic process	3.57E-02	**1.14E-01**	6.71	2/153	36/18493
GO:0006693	prostaglandin metabolic process	3.57E-02	**1.14E-01**	6.71	2/153	36/18493
GO:0042092	type 2 immune response	3.57E-02	**1.14E-01**	6.71	2/153	36/18493
GO:0090279	regulation of calcium ion import	3.57E-02	**1.14E-01**	6.71	2/153	36/18493
GO:0000187	activation of MAPK activity	3.61E-02	**1.15E-01**	3.22	4/153	150/18493
GO:0008360	regulation of cell shape	3.69E-02	**1.17E-01**	3.20	4/153	151/18493
GO:0002823	negative regulation of adaptive immune response based on somatic recombination of immune receptors built from immunoglobulin superfamily domains	3.75E-02	**1.18E-01**	6.53	2/153	37/18493
GO:0014912	negative regulation of smooth muscle cell migration	3.75E-02	**1.18E-01**	6.53	2/153	37/18493
GO:0048846	axon extension involved in axon guidance	3.75E-02	**1.18E-01**	6.53	2/153	37/18493
GO:0070741	response to interleukin-6	3.75E-02	**1.18E-01**	6.53	2/153	37/18493
GO:0071276	cellular response to cadmium ion	3.75E-02	**1.18E-01**	6.53	2/153	37/18493
GO:1902284	neuron projection extension involved in neuron projection guidance	3.75E-02	**1.18E-01**	6.53	2/153	37/18493
GO:0000302	response to reactive oxygen species	3.87E-02	**1.22E-01**	2.70	5/153	224/18493
GO:0048145	regulation of fibroblast proliferation	3.87E-02	**1.22E-01**	4.03	3/153	90/18493
GO:0002714	positive regulation of B cell mediated immunity	3.94E-02	**1.24E-01**	6.36	2/153	38/18493
GO:0002891	positive regulation of immunoglobulin mediated immune response	3.94E-02	**1.24E-01**	6.36	2/153	38/18493
GO:0030204	chondroitin sulfate metabolic process	3.94E-02	**1.24E-01**	6.36	2/153	38/18493
GO:0048010	vascular endothelial growth factor receptor signaling pathway	3.98E-02	**1.24E-01**	3.98	3/153	91/18493
GO:0048144	fibroblast proliferation	3.98E-02	**1.24E-01**	3.98	3/153	91/18493
GO:0090630	activation of GTPase activity	3.98E-02	**1.24E-01**	3.98	3/153	91/18493
GO:1901570	fatty acid derivative biosynthetic process	3.98E-02	**1.24E-01**	3.98	3/153	91/18493
GO:0060560	developmental growth involved in morphogenesis	4.06E-02	**1.27E-01**	2.66	5/153	227/18493

GO:0051896	regulation of protein kinase B signaling	4.12E-02	**1.28E-01**	2.65	5/153	228/18493
GO:1902229	regulation of intrinsic apoptotic signaling pathway in response to DNA damage	4.13E-02	**1.28E-01**	6.20	2/153	39/18493
GO:0030203	glycosaminoglycan metabolic process	4.16E-02	**1.29E-01**	3.08	4/153	157/18493
GO:0071375	cellular response to peptide hormone stimulus	4.26E-02	**1.32E-01**	2.36	6/153	307/18493
GO:1903426	regulation of reactive oxygen species biosynthetic process	4.31E-02	**1.33E-01**	3.86	3/153	94/18493
GO:0030225	macrophage differentiation	4.32E-02	**1.33E-01**	6.04	2/153	40/18493
GO:0097028	dendritic cell differentiation	4.32E-02	**1.33E-01**	6.04	2/153	40/18493
GO:0006644	phospholipid metabolic process	4.32E-02	**1.33E-01**	2.04	8/153	473/18493
GO:0022604	regulation of cell morphogenesis	4.37E-02	**1.35E-01**	2.04	8/153	474/18493
GO:0002367	cytokine production involved in immune response	4.43E-02	**1.36E-01**	3.82	3/153	95/18493
GO:0016485	protein processing	4.43E-02	**1.36E-01**	2.34	6/153	310/18493
GO:0035821	modification of morphology or physiology of other organism	4.49E-02	**1.38E-01**	3.00	4/153	161/18493
GO:1990138	neuron projection extension	4.49E-02	**1.38E-01**	3.00	4/153	161/18493
GO:0030866	cortical actin cytoskeleton organization	4.52E-02	**1.38E-01**	5.90	2/153	41/18493
GO:0010771	negative regulation of cell morphogenesis involved in differentiation	4.54E-02	**1.39E-01**	3.78	3/153	96/18493
GO:0043902	positive regulation of multi-organism process	4.57E-02	**1.40E-01**	2.98	4/153	162/18493
GO:0032872	regulation of stress-activated MAPK cascade	4.59E-02	**1.40E-01**	2.57	5/153	235/18493
GO:0001666	response to hypoxia	4.66E-02	**1.42E-01**	2.31	6/153	314/18493
GO:0001953	negative regulation of cell-matrix adhesion	4.72E-02	**1.43E-01**	5.76	2/153	42/18493
GO:0002437	inflammatory response to antigenic stimulus	4.72E-02	**1.43E-01**	5.76	2/153	42/18493
GO:0032459	regulation of protein oligomerization	4.72E-02	**1.43E-01**	5.76	2/153	42/18493
GO:0043124	negative regulation of I-kappaB kinase/NF-kappaB signaling	4.72E-02	**1.43E-01**	5.76	2/153	42/18493
GO:0045581	negative regulation of T cell differentiation	4.72E-02	**1.43E-01**	5.76	2/153	42/18493
GO:0070302	regulation of stress-activated protein kinase signaling cascade	4.73E-02	**1.43E-01**	2.55	5/153	237/18493
GO:0048015	phosphatidylinositol-mediated signaling	4.75E-02	**1.43E-01**	2.95	4/153	164/18493
GO:1903829	positive regulation of cellular protein localization	4.91E-02	**1.47E-01**	2.28	6/153	318/18493

GO:2000045	regulation of G1/S transition of mitotic cell cycle	4.92E-02	**1.47E-01**	2.91	4/153	166/18493
GO:0002534	cytokine production involved in inflammatory response	4.92E-02	**1.47E-01**	5.62	2/153	43/18493
GO:0042771	intrinsic apoptotic signaling pathway in response to DNA damage by p53 class mediator	4.92E-02	**1.47E-01**	5.62	2/153	43/18493
GO:0050654	chondroitin sulfate proteoglycan metabolic process	4.92E-02	**1.47E-01**	5.62	2/153	43/18493
GO:1900087	positive regulation of G1/S transition of mitotic cell cycle	4.92E-02	**1.47E-01**	5.62	2/153	43/18493
GO:1901658	glycosyl compound catabolic process	4.92E-02	**1.47E-01**	5.62	2/153	43/18493
GO:1902667	regulation of axon guidance	4.92E-02	**1.47E-01**	5.62	2/153	43/18493
GO:0042742	defense response to bacterium	4.97E-02	**1.48E-01**	2.27	6/153	319/18493
GO:0006022	aminoglycan metabolic process	5.01E-02	**1.49E-01**	2.90	4/153	167/18493
GO:0048017	inositol lipid-mediated signaling	5.01E-02	**1.49E-01**	2.90	4/153	167/18493
GO:0050848	regulation of calcium-mediated signaling	5.02E-02	**1.49E-01**	3.63	3/153	100/18493
GO:0120034	positive regulation of plasma membrane bounded cell projection assembly	5.02E-02	**1.49E-01**	3.63	3/153	100/18493
GO:0046394	carboxylic acid biosynthetic process	4.09E-03	**1.25E-01**	8.98	3/15	412/18493
GO:0016053	organic acid biosynthetic process	4.12E-03	**1.25E-01**	8.96	3/15	413/18493
GO:0046718	viral entry into host cell	4.22E-03	**1.25E-01**	20.38	2/15	121/18493
GO:0090101	negative regulation of transmembrane receptor protein serine/threonine kinase signaling pathway	4.35E-03	**1.25E-01**	20.05	2/15	123/18493
GO:0044282	small molecule catabolic process	4.64E-03	**1.25E-01**	8.58	3/15	431/18493
GO:0030260	entry into host cell	5.07E-03	**1.25E-01**	18.54	2/15	133/18493
GO:0034620	cellular response to unfolded protein	5.07E-03	**1.25E-01**	18.54	2/15	133/18493
GO:0044409	entry into host	5.07E-03	**1.25E-01**	18.54	2/15	133/18493
GO:0051806	entry into cell of other organism involved in symbiotic interaction	5.07E-03	**1.25E-01**	18.54	2/15	133/18493
GO:0051828	entry into other organism involved in symbiotic interaction	5.07E-03	**1.25E-01**	18.54	2/15	133/18493
GO:0009408	response to heat	6.00E-03	**1.25E-01**	17.01	2/15	145/18493
GO:0051092	positive regulation of NF-kappaB transcription factor activity	6.08E-03	**1.25E-01**	16.89	2/15	146/18493
GO:0035967	cellular response to topologically incorrect protein	6.49E-03	**1.25E-01**	16.33	2/15	151/18493

GO:0090288	negative regulation of cellular response to growth factor stimulus	7.25E-03	**1.25E-01**	15.41	2/15	160/18493
GO:0006986	response to unfolded protein	8.06E-03	**1.25E-01**	14.59	2/15	169/18493
GO:0042989	sequestering of actin monomers	8.08E-03	**1.25E-01**	123.29	1/15	10/18493
GO:0002003	angiotensin maturation	8.89E-03	**1.25E-01**	112.08	1/15	11/18493
GO:0010172	embryonic body morphogenesis	8.89E-03	**1.25E-01**	112.08	1/15	11/18493
GO:0016322	neuron remodeling	8.89E-03	**1.25E-01**	112.08	1/15	11/18493
GO:0018065	protein-cofactor linkage	8.89E-03	**1.25E-01**	112.08	1/15	11/18493
GO:0033632	regulation of cell-cell adhesion mediated by integrin	8.89E-03	**1.25E-01**	112.08	1/15	11/18493
GO:0090084	negative regulation of inclusion body assembly	8.89E-03	**1.25E-01**	112.08	1/15	11/18493
GO:0002002	regulation of angiotensin levels in blood	9.69E-03	**1.25E-01**	102.74	1/15	12/18493
GO:0035641	locomotory exploration behavior	9.69E-03	**1.25E-01**	102.74	1/15	12/18493
GO:0097201	negative regulation of transcription from RNA polymerase II promoter in response to stress	9.69E-03	**1.25E-01**	102.74	1/15	12/18493
GO:0035966	response to topologically incorrect protein	1.00E-02	**1.25E-01**	13.05	2/15	189/18493
GO:0006694	steroid biosynthetic process	1.04E-02	**1.25E-01**	12.78	2/15	193/18493
GO:0009086	methionine biosynthetic process	1.05E-02	**1.25E-01**	94.84	1/15	13/18493
GO:0032530	regulation of microvillus organization	1.05E-02	**1.25E-01**	94.84	1/15	13/18493
GO:0032536	regulation of cell projection size	1.05E-02	**1.25E-01**	94.84	1/15	13/18493
GO:0070431	nucleotide-binding oligomerization domain containing 2 signaling pathway	1.05E-02	**1.25E-01**	94.84	1/15	13/18493
GO:0071872	cellular response to epinephrine stimulus	1.05E-02	**1.25E-01**	94.84	1/15	13/18493
GO:1903054	negative regulation of extracellular matrix organization	1.05E-02	**1.25E-01**	94.84	1/15	13/18493
GO:0007626	locomotory behavior	1.05E-02	**1.25E-01**	12.71	2/15	194/18493
GO:0000098	sulfur amino acid catabolic process	1.13E-02	**1.25E-01**	88.06	1/15	14/18493
GO:0050667	homocysteine metabolic process	1.13E-02	**1.25E-01**	88.06	1/15	14/18493
GO:0090399	replicative senescence	1.13E-02	**1.25E-01**	88.06	1/15	14/18493
GO:0099558	maintenance of synapse structure	1.13E-02	**1.25E-01**	88.06	1/15	14/18493
GO:1901386	negative regulation of voltage-gated calcium channel activity	1.13E-02	**1.25E-01**	88.06	1/15	14/18493

GO:0009266	response to temperature stimulus	1.20E-02	**1.25E-01**	11.85	2/15	208/18493
GO:0032271	regulation of protein polymerization	1.20E-02	**1.25E-01**	11.85	2/15	208/18493
GO:0010715	regulation of extracellular matrix disassembly	1.21E-02	**1.25E-01**	82.19	1/15	15/18493
GO:0033540	fatty acid beta-oxidation using acyl-CoA oxidase	1.21E-02	**1.25E-01**	82.19	1/15	15/18493
GO:0033631	cell-cell adhesion mediated by integrin	1.21E-02	**1.25E-01**	82.19	1/15	15/18493
GO:0051701	interaction with host	1.21E-02	**1.25E-01**	11.80	2/15	209/18493
GO:0071871	response to epinephrine	1.29E-02	**1.25E-01**	77.05	1/15	16/18493
GO:0090083	regulation of inclusion body assembly	1.29E-02	**1.25E-01**	77.05	1/15	16/18493
GO:0006457	protein folding	1.31E-02	**1.25E-01**	11.31	2/15	218/18493
GO:0042445	hormone metabolic process	1.31E-02	**1.25E-01**	11.31	2/15	218/18493
GO:2001234	negative regulation of apoptotic signaling pathway	1.36E-02	**1.25E-01**	11.11	2/15	222/18493
GO:0006555	methionine metabolic process	1.37E-02	**1.25E-01**	72.52	1/15	17/18493
GO:0006704	glucocorticoid biosynthetic process	1.45E-02	**1.25E-01**	68.49	1/15	18/18493
GO:0010224	response to UV-B	1.45E-02	**1.25E-01**	68.49	1/15	18/18493
GO:1905065	positive regulation of vascular smooth muscle cell differentiation	1.45E-02	**1.25E-01**	68.49	1/15	18/18493
GO:0090092	regulation of transmembrane receptor protein serine/threonine kinase signaling pathway	1.51E-02	**1.25E-01**	10.49	2/15	235/18493
GO:0001991	regulation of systemic arterial blood pressure by circulatory renin-angiotensin	1.53E-02	**1.25E-01**	64.89	1/15	19/18493
GO:0006534	cysteine metabolic process	1.53E-02	**1.25E-01**	64.89	1/15	19/18493
GO:0051016	barbed-end actin filament capping	1.53E-02	**1.25E-01**	64.89	1/15	19/18493
GO:1901673	regulation of mitotic spindle assembly	1.53E-02	**1.25E-01**	64.89	1/15	19/18493
GO:1902236	negative regulation of endoplasmic reticulum stress-induced intrinsic apoptotic signaling pathway	1.53E-02	**1.25E-01**	64.89	1/15	19/18493
GO:0051131	chaperone-mediated protein complex assembly	1.61E-02	**1.25E-01**	61.64	1/15	20/18493
GO:0097242	amyloid-beta clearance	1.61E-02	**1.25E-01**	61.64	1/15	20/18493
GO:0000097	sulfur amino acid biosynthetic process	1.69E-02	**1.25E-01**	58.71	1/15	21/18493
GO:0009070	serine family amino acid biosynthetic process	1.69E-02	**1.25E-01**	58.71	1/15	21/18493
GO:0046034	ATP metabolic process	1.70E-02	**1.25E-01**	9.86	2/15	250/18493

GO:0051091	positive regulation of DNA-binding transcription factor activity	1.77E-02	**1.25E-01**	9.67	2/15	255/18493
GO:0032069	regulation of nuclease activity	1.77E-02	**1.25E-01**	56.04	1/15	22/18493
GO:0070841	inclusion body assembly	1.77E-02	**1.25E-01**	56.04	1/15	22/18493
GO:0009067	aspartate family amino acid biosynthetic process	1.85E-02	**1.25E-01**	53.60	1/15	23/18493
GO:0010226	response to lithium ion	1.85E-02	**1.25E-01**	53.60	1/15	23/18493
GO:0034976	response to endoplasmic reticulum stress	1.89E-02	**1.25E-01**	9.34	2/15	264/18493
GO:0016054	organic acid catabolic process	1.90E-02	**1.25E-01**	9.30	2/15	265/18493
GO:0046395	carboxylic acid catabolic process	1.90E-02	**1.25E-01**	9.30	2/15	265/18493
GO:0032528	microvillus organization	1.93E-02	**1.25E-01**	51.37	1/15	24/18493
GO:0035640	exploration behavior	1.93E-02	**1.25E-01**	51.37	1/15	24/18493
GO:1900120	regulation of receptor binding	1.93E-02	**1.25E-01**	51.37	1/15	24/18493
GO:0006735	NADH regeneration	2.01E-02	**1.25E-01**	49.31	1/15	25/18493
GO:0008211	glucocorticoid metabolic process	2.01E-02	**1.25E-01**	49.31	1/15	25/18493
GO:0009404	toxin metabolic process	2.01E-02	**1.25E-01**	49.31	1/15	25/18493
GO:0031116	positive regulation of microtubule polymerization	2.01E-02	**1.25E-01**	49.31	1/15	25/18493
GO:0061621	canonical glycolysis	2.01E-02	**1.25E-01**	49.31	1/15	25/18493
GO:0061718	glucose catabolic process to pyruvate	2.01E-02	**1.25E-01**	49.31	1/15	25/18493
GO:0051258	protein polymerization	2.04E-02	**1.25E-01**	8.97	2/15	275/18493
GO:0003081	regulation of systemic arterial blood pressure by renin-angiotensin	2.09E-02	**1.25E-01**	47.42	1/15	26/18493
GO:0042026	protein refolding	2.09E-02	**1.25E-01**	47.42	1/15	26/18493
GO:0051152	positive regulation of smooth muscle cell differentiation	2.09E-02	**1.25E-01**	47.42	1/15	26/18493
GO:0061615	glycolytic process through fructose-6-phosphate	2.09E-02	**1.25E-01**	47.42	1/15	26/18493
GO:0061620	glycolytic process through glucose-6-phosphate	2.09E-02	**1.25E-01**	47.42	1/15	26/18493
GO:0090169	regulation of spindle assembly	2.09E-02	**1.25E-01**	47.42	1/15	26/18493
GO:0009205	purine ribonucleoside triphosphate metabolic process	2.11E-02	**1.25E-01**	8.81	2/15	280/18493
GO:0090287	regulation of cellular response to growth factor stimulus	2.13E-02	**1.25E-01**	8.74	2/15	282/18493
GO:0044550	secondary metabolite biosynthetic process	2.17E-02	**1.25E-01**	45.66	1/15	27/18493
GO:0009167	purine ribonucleoside monophosphate metabolic process	2.19E-02	**1.25E-01**	8.62	2/15	286/18493

GO:0009199	ribonucleoside triphosphate metabolic process	2.19E-02	**1.25E-01**	8.62	2/15	286/18493
GO:0009126	purine nucleoside monophosphate metabolic process	2.21E-02	**1.25E-01**	8.59	2/15	287/18493
GO:0009144	purine nucleoside triphosphate metabolic process	2.21E-02	**1.25E-01**	8.59	2/15	287/18493
GO:0019058	viral life cycle	2.23E-02	**1.25E-01**	8.53	2/15	289/18493
GO:0007020	microtubule nucleation	2.25E-02	**1.25E-01**	44.03	1/15	28/18493
GO:0072330	monocarboxylic acid biosynthetic process	2.29E-02	**1.25E-01**	8.42	2/15	293/18493
GO:0031112	positive regulation of microtubule polymerization or depolymerization	2.33E-02	**1.25E-01**	42.51	1/15	29/18493
GO:0009161	ribonucleoside monophosphate metabolic process	2.40E-02	**1.25E-01**	8.22	2/15	300/18493
GO:1902235	regulation of endoplasmic reticulum stress-induced intrinsic apoptotic signaling pathway	2.41E-02	**1.25E-01**	41.10	1/15	30/18493
GO:0045648	positive regulation of erythrocyte differentiation	2.49E-02	**1.25E-01**	39.77	1/15	31/18493
GO:0051085	chaperone cofactor-dependent protein refolding	2.49E-02	**1.25E-01**	39.77	1/15	31/18493
GO:1905063	regulation of vascular smooth muscle cell differentiation	2.49E-02	**1.25E-01**	39.77	1/15	31/18493
GO:0009141	nucleoside triphosphate metabolic process	2.50E-02	**1.25E-01**	8.03	2/15	307/18493
GO:0006007	glucose catabolic process	2.57E-02	**1.25E-01**	38.53	1/15	32/18493
GO:0016486	peptide hormone processing	2.57E-02	**1.25E-01**	38.53	1/15	32/18493
GO:0001666	response to hypoxia	2.61E-02	**1.25E-01**	7.85	2/15	314/18493
GO:0033198	response to ATP	2.64E-02	**1.25E-01**	37.36	1/15	33/18493
GO:0008202	steroid metabolic process	2.67E-02	**1.25E-01**	7.75	2/15	318/18493
GO:0071868	cellular response to monoamine stimulus	2.72E-02	**1.25E-01**	36.26	1/15	34/18493
GO:0071870	cellular response to catecholamine stimulus	2.72E-02	**1.25E-01**	36.26	1/15	34/18493
GO:0009123	nucleoside monophosphate metabolic process	2.73E-02	**1.25E-01**	7.66	2/15	322/18493
GO:0036293	response to decreased oxygen levels	2.78E-02	**1.25E-01**	7.59	2/15	325/18493
GO:0070423	nucleotide-binding oligomerization domain containing signaling pathway	2.80E-02	**1.25E-01**	35.22	1/15	35/18493
GO:1901020	negative regulation of calcium ion transmembrane transporter activity	2.80E-02	**1.25E-01**	35.22	1/15	35/18493
GO:1901099	negative regulation of signal transduction in absence of ligand	2.80E-02	**1.25E-01**	35.22	1/15	35/18493

GO:2001240	negative regulation of extrinsic apoptotic signaling pathway in absence of ligand	2.80E-02	**1.25E-01**	35.22	1/15	35/18493
GO:0001662	behavioral fear response	2.88E-02	**1.25E-01**	34.25	1/15	36/18493
GO:0002209	behavioral defense response	2.88E-02	**1.25E-01**	34.25	1/15	36/18493
GO:0006699	bile acid biosynthetic process	2.88E-02	**1.25E-01**	34.25	1/15	36/18493
GO:0010591	regulation of lamellipodium assembly	2.88E-02	**1.25E-01**	34.25	1/15	36/18493
GO:0033120	positive regulation of RNA splicing	2.88E-02	**1.25E-01**	34.25	1/15	36/18493
GO:0035872	nucleotide-binding domain, leucine rich repeat containing receptor signaling pathway	2.88E-02	**1.25E-01**	34.25	1/15	36/18493
GO:0051084	'de novo' posttranslational protein folding	2.88E-02	**1.25E-01**	34.25	1/15	36/18493
GO:0060236	regulation of mitotic spindle organization	2.88E-02	**1.25E-01**	34.25	1/15	36/18493
GO:1900115	extracellular regulation of signal transduction	2.88E-02	**1.25E-01**	34.25	1/15	36/18493
GO:1900116	extracellular negative regulation of signal transduction	2.88E-02	**1.25E-01**	34.25	1/15	36/18493
GO:1901385	regulation of voltage-gated calcium channel activity	2.88E-02	**1.25E-01**	34.25	1/15	36/18493
GO:0050435	amyloid-beta metabolic process	2.96E-02	**1.25E-01**	33.32	1/15	37/18493
GO:0051693	actin filament capping	2.96E-02	**1.25E-01**	33.32	1/15	37/18493
GO:0007178	transmembrane receptor protein serine/threonine kinase signaling pathway	3.02E-02	**1.25E-01**	7.25	2/15	340/18493
GO:0006734	NADH metabolic process	3.04E-02	**1.25E-01**	32.44	1/15	38/18493
GO:0042596	fear response	3.04E-02	**1.25E-01**	32.44	1/15	38/18493
GO:0071867	response to monoamine	3.04E-02	**1.25E-01**	32.44	1/15	38/18493
GO:0071869	response to catecholamine	3.04E-02	**1.25E-01**	32.44	1/15	38/18493
GO:1902903	regulation of supramolecular fiber organization	3.07E-02	**1.25E-01**	7.19	2/15	343/18493
GO:1903053	regulation of extracellular matrix organization	3.12E-02	**1.25E-01**	31.61	1/15	39/18493
GO:0070482	response to oxygen levels	3.17E-02	**1.25E-01**	7.07	2/15	349/18493
GO:0001990	regulation of systemic arterial blood pressure by hormone	3.20E-02	**1.25E-01**	30.82	1/15	40/18493
GO:0006458	'de novo' protein folding	3.20E-02	**1.25E-01**	30.82	1/15	40/18493
GO:0030835	negative regulation of actin filament depolymerization	3.20E-02	**1.25E-01**	30.82	1/15	40/18493
GO:0090224	regulation of spindle organization	3.20E-02	**1.25E-01**	30.82	1/15	40/18493

GO:1903170	negative regulation of calcium ion transmembrane transport	3.20E-02	**1.25E-01**	30.82	1/15	40/18493
GO:0035886	vascular smooth muscle cell differentiation	3.28E-02	**1.26E-01**	30.07	1/15	41/18493
GO:1901028	regulation of mitochondrial outer membrane permeabilization involved in apoptotic signaling pathway	3.28E-02	**1.26E-01**	30.07	1/15	41/18493
GO:0042551	neuron maturation	3.35E-02	**1.27E-01**	29.35	1/15	42/18493
GO:0007212	dopamine receptor signaling pathway	3.43E-02	**1.27E-01**	28.67	1/15	43/18493
GO:0010613	positive regulation of cardiac muscle hypertrophy	3.43E-02	**1.27E-01**	28.67	1/15	43/18493
GO:0014742	positive regulation of muscle hypertrophy	3.43E-02	**1.27E-01**	28.67	1/15	43/18493
GO:0031113	regulation of microtubule polymerization	3.43E-02	**1.27E-01**	28.67	1/15	43/18493
GO:1903573	negative regulation of response to endoplasmic reticulum stress	3.43E-02	**1.27E-01**	28.67	1/15	43/18493
GO:0032535	regulation of cellular component size	3.48E-02	**1.27E-01**	6.72	2/15	367/18493
GO:0000096	sulfur amino acid metabolic process	3.51E-02	**1.27E-01**	28.02	1/15	44/18493
GO:0033628	regulation of cell adhesion mediated by integrin	3.51E-02	**1.27E-01**	28.02	1/15	44/18493
GO:0009069	serine family amino acid metabolic process	3.59E-02	**1.28E-01**	27.40	1/15	45/18493
GO:1900034	regulation of cellular response to heat	3.59E-02	**1.28E-01**	27.40	1/15	45/18493
GO:0001961	positive regulation of cytokine-mediated signaling pathway	3.67E-02	**1.28E-01**	26.80	1/15	46/18493
GO:0008206	bile acid metabolic process	3.67E-02	**1.28E-01**	26.80	1/15	46/18493
GO:0032757	positive regulation of interleukin-8 production	3.67E-02	**1.28E-01**	26.80	1/15	46/18493
GO:2001239	regulation of extrinsic apoptotic signaling pathway in absence of ligand	3.67E-02	**1.28E-01**	26.80	1/15	46/18493
GO:0045646	regulation of erythrocyte differentiation	3.75E-02	**1.30E-01**	26.23	1/15	47/18493
GO:0010639	negative regulation of organelle organization	3.78E-02	**1.30E-01**	6.42	2/15	384/18493
GO:1902743	regulation of lamellipodium organization	3.82E-02	**1.31E-01**	25.68	1/15	48/18493
GO:0003044	regulation of systemic arterial blood pressure mediated by a chemical signal	3.90E-02	**1.32E-01**	25.16	1/15	49/18493
GO:2001233	regulation of apoptotic signaling pathway	3.90E-02	**1.32E-01**	6.31	2/15	391/18493
GO:0010171	body morphogenesis	3.98E-02	**1.33E-01**	24.66	1/15	50/18493
GO:0030834	regulation of actin filament depolymerization	3.98E-02	**1.33E-01**	24.66	1/15	50/18493

GO:0009066	aspartate family amino acid metabolic process	4.06E-02	**1.33E-01**	24.17	1/15	51/18493
GO:0030514	negative regulation of BMP signaling pathway	4.06E-02	**1.33E-01**	24.17	1/15	51/18493
GO:0097345	mitochondrial outer membrane permeabilization	4.06E-02	**1.33E-01**	24.17	1/15	51/18493
GO:2001258	negative regulation of cation channel activity	4.06E-02	**1.33E-01**	24.17	1/15	51/18493
GO:0051150	regulation of smooth muscle cell differentiation	4.14E-02	**1.33E-01**	23.71	1/15	52/18493
GO:0060760	positive regulation of response to cytokine stimulus	4.14E-02	**1.33E-01**	23.71	1/15	52/18493
GO:0009435	NAD biosynthetic process	4.22E-02	**1.33E-01**	23.26	1/15	53/18493
GO:0010823	negative regulation of mitochondrion organization	4.22E-02	**1.33E-01**	23.26	1/15	53/18493
GO:0030042	actin filament depolymerization	4.22E-02	**1.33E-01**	23.26	1/15	53/18493
GO:0043954	cellular component maintenance	4.22E-02	**1.33E-01**	23.26	1/15	53/18493
GO:0019320	hexose catabolic process	4.29E-02	**1.33E-01**	22.83	1/15	54/18493
GO:0031295	T cell costimulation	4.29E-02	**1.33E-01**	22.83	1/15	54/18493
GO:0051090	regulation of DNA-binding transcription factor activity	4.29E-02	**1.33E-01**	5.98	2/15	412/18493
GO:0031294	lymphocyte costimulation	4.37E-02	**1.35E-01**	22.42	1/15	55/18493
GO:0044273	sulfur compound catabolic process	4.45E-02	**1.37E-01**	22.02	1/15	56/18493
GO:0030837	negative regulation of actin filament polymerization	4.53E-02	**1.38E-01**	21.63	1/15	57/18493
GO:1902110	positive regulation of mitochondrial membrane permeability involved in apoptotic process	4.53E-02	**1.38E-01**	21.63	1/15	57/18493
GO:0010803	regulation of tumor necrosis factor-mediated signaling pathway	4.68E-02	**1.40E-01**	20.90	1/15	59/18493
GO:0070059	intrinsic apoptotic signaling pathway in response to endoplasmic reticulum stress	4.68E-02	**1.40E-01**	20.90	1/15	59/18493
GO:1902686	mitochondrial outer membrane permeabilization involved in programmed cell death	4.68E-02	**1.40E-01**	20.90	1/15	59/18493
GO:0002753	cytoplasmic pattern recognition receptor signaling pathway	4.76E-02	**1.40E-01**	20.55	1/15	60/18493
GO:0061077	chaperone-mediated protein folding	4.76E-02	**1.40E-01**	20.55	1/15	60/18493
GO:0090307	mitotic spindle assembly	4.76E-02	**1.40E-01**	20.55	1/15	60/18493
GO:0035794	positive regulation of mitochondrial membrane permeability	4.84E-02	**1.42E-01**	20.21	1/15	61/18493
GO:0019748	secondary metabolic process	4.91E-02	**1.43E-01**	19.88	1/15	62/18493

GO:1902108	regulation of mitochondrial membrane permeability involved in apoptotic process	4.99E-02	**1.44E-01**	19.57	1/15	63/18493
GO:1905710	positive regulation of membrane permeability	4.99E-02	**1.44E-01**	19.57	1/15	63/18493
GO:0043254	regulation of protein complex assembly	5.05E-02	**1.44E-01**	5.47	2/15	451/18493
GO:0046365	monosaccharide catabolic process	5.07E-02	**1.44E-01**	19.26	1/15	64/18493
GO:1901880	negative regulation of protein depolymerization	5.07E-02	**1.44E-01**	19.26	1/15	64/18493
GO:0033627	cell adhesion mediated by integrin	5.15E-02	**1.44E-01**	18.97	1/15	65/18493
GO:0051926	negative regulation of calcium ion transport	5.15E-02	**1.44E-01**	18.97	1/15	65/18493
GO:0032677	regulation of interleukin-8 production	5.22E-02	**1.44E-01**	18.68	1/15	66/18493
GO:0030032	lamellipodium assembly	5.30E-02	**1.44E-01**	18.40	1/15	67/18493
GO:0006625	protein targeting to peroxisome	5.38E-02	**1.44E-01**	18.13	1/15	68/18493
GO:0072662	protein localization to peroxisome	5.38E-02	**1.44E-01**	18.13	1/15	68/18493
GO:0072663	establishment of protein localization to peroxisome	5.38E-02	**1.44E-01**	18.13	1/15	68/18493
GO:1901607	alpha-amino acid biosynthetic process	5.38E-02	**1.44E-01**	18.13	1/15	68/18493
GO:0043574	peroxisomal transport	5.45E-02	**1.44E-01**	17.87	1/15	69/18493
GO:0032272	negative regulation of protein polymerization	5.53E-02	**1.44E-01**	17.61	1/15	70/18493
GO:1901264	carbohydrate derivative transport	5.53E-02	**1.44E-01**	17.61	1/15	70/18493
GO:0019674	NAD metabolic process	5.61E-02	**1.44E-01**	17.36	1/15	71/18493
GO:0031110	regulation of microtubule polymerization or depolymerization	5.61E-02	**1.44E-01**	17.36	1/15	71/18493
GO:0038034	signal transduction in absence of ligand	5.61E-02	**1.44E-01**	17.36	1/15	71/18493
GO:0043242	negative regulation of protein complex disassembly	5.61E-02	**1.44E-01**	17.36	1/15	71/18493
GO:0046785	microtubule polymerization	5.61E-02	**1.44E-01**	17.36	1/15	71/18493
GO:0097192	extrinsic apoptotic signaling pathway in absence of ligand	5.61E-02	**1.44E-01**	17.36	1/15	71/18493
GO:0006635	fatty acid beta-oxidation	5.69E-02	**1.44E-01**	17.12	1/15	72/18493
GO:0033555	multicellular organismal response to stress	5.69E-02	**1.44E-01**	17.12	1/15	72/18493
GO:0043312	neutrophil degranulation	5.75E-02	**1.44E-01**	5.08	2/15	485/18493
GO:0031397	negative regulation of protein ubiquitination	5.76E-02	**1.44E-01**	16.89	1/15	73/18493
GO:0032637	interleukin-8 production	5.76E-02	**1.44E-01**	16.89	1/15	73/18493
GO:0046902	regulation of mitochondrial membrane permeability	5.76E-02	**1.44E-01**	16.89	1/15	73/18493

GO:0002283	neutrophil activation involved in immune response	5.82E-02	**1.44E-01**	5.05	2/15	488/18493
GO:0009150	purine ribonucleotide metabolic process	5.82E-02	**1.44E-01**	5.05	2/15	488/18493
GO:0006096	glycolytic process	5.84E-02	**1.44E-01**	16.66	1/15	74/18493
GO:0010611	regulation of cardiac muscle hypertrophy	5.84E-02	**1.44E-01**	16.66	1/15	74/18493
GO:0022617	extracellular matrix disassembly	5.84E-02	**1.44E-01**	16.66	1/15	74/18493
GO:0032507	maintenance of protein location in cell	5.84E-02	**1.44E-01**	16.66	1/15	74/18493
GO:0006757	ATP generation from ADP	5.92E-02	**1.44E-01**	16.44	1/15	75/18493
GO:0043618	regulation of transcription from RNA polymerase II promoter in response to stress	5.92E-02	**1.44E-01**	16.44	1/15	75/18493
GO:0051051	negative regulation of transport	5.97E-02	**1.44E-01**	4.98	2/15	495/18493
GO:0014743	regulation of muscle hypertrophy	5.99E-02	**1.44E-01**	16.22	1/15	76/18493
GO:0051145	smooth muscle cell differentiation	5.99E-02	**1.44E-01**	16.22	1/15	76/18493
GO:0090066	regulation of anatomical structure size	6.01E-02	**1.44E-01**	4.96	2/15	497/18493
GO:0042119	neutrophil activation	6.03E-02	**1.44E-01**	4.95	2/15	498/18493
GO:0002446	neutrophil mediated immunity	6.05E-02	**1.44E-01**	4.94	2/15	499/18493
GO:0032413	negative regulation of ion transmembrane transporter activity	6.07E-02	**1.44E-01**	16.01	1/15	77/18493
GO:0034644	cellular response to UV	6.15E-02	**1.44E-01**	15.81	1/15	78/18493
GO:0042866	pyruvate biosynthetic process	6.15E-02	**1.44E-01**	15.81	1/15	78/18493
GO:0051289	protein homotetramerization	6.15E-02	**1.44E-01**	15.81	1/15	78/18493
GO:1905897	regulation of response to endoplasmic reticulum stress	6.15E-02	**1.44E-01**	15.81	1/15	78/18493
GO:0030512	negative regulation of transforming growth factor beta receptor signaling pathway	6.22E-02	**1.45E-01**	15.61	1/15	79/18493
GO:1901879	regulation of protein depolymerization	6.22E-02	**1.45E-01**	15.61	1/15	79/18493
GO:0007031	peroxisome organization	6.37E-02	**1.45E-01**	15.22	1/15	81/18493
GO:0032436	positive regulation of proteasomal ubiquitin-dependent protein catabolic process	6.37E-02	**1.45E-01**	15.22	1/15	81/18493
GO:0043620	regulation of DNA-templated transcription in response to stress	6.37E-02	**1.45E-01**	15.22	1/15	81/18493
GO:0090559	regulation of membrane permeability	6.37E-02	**1.45E-01**	15.22	1/15	81/18493

GO:1903845	negative regulation of cellular response to transforming growth factor beta stimulus	6.37E-02	**1.45E-01**	15.22	1/15	81/18493
GO:1903321	negative regulation of protein modification by small protein conjugation or removal	6.53E-02	**1.47E-01**	14.85	1/15	83/18493
GO:0042446	hormone biosynthetic process	6.60E-02	**1.47E-01**	14.68	1/15	84/18493
GO:0046031	ADP metabolic process	6.60E-02	**1.47E-01**	14.68	1/15	84/18493
GO:0050886	endocrine process	6.60E-02	**1.47E-01**	14.68	1/15	84/18493
GO:0097581	lamellipodium organization	6.60E-02	**1.47E-01**	14.68	1/15	84/18493
GO:0008652	cellular amino acid biosynthetic process	6.68E-02	**1.49E-01**	14.50	1/15	85/18493
GO:1901019	regulation of calcium ion transmembrane transporter activity	6.76E-02	**1.50E-01**	14.34	1/15	86/18493

Table S5. Logistic regression results measuring association between *PVRIG* groups and functional gene sets

Variables			Total (N)	PVRIG-High (n)	Statistic	p-value	log(OR) (CI)	OR
		Bindea-T_cells	602	301	11.56	**6.73E-31**	1.68 (1.4-1.97)	5.35
		Bindea-B_cells	602	301	11.01	**3.39E-28**	1.48 (1.23-1.76)	4.41
		Bindea-Cytotoxic_cells	602	301	10.92	**8.86E-28**	1.5 (1.24-1.78)	4.48
		Bindea-Th1_cells	602	301	9.66	**4.27E-22**	1.14 (0.92-1.38)	3.13
		Bindea-Tgd	602	301	9.58	**9.56E-22**	1.07 (0.86-1.29)	2.91
		Bindea-aDC	602	301	8.98	**2.79E-19**	1.01 (0.79-1.23)	2.74
		Bindea-Tem	602	301	8.29	**1.18E-16**	0.85 (0.66-1.06)	2.34
		Bindea-TFH	602	301	8.2	**2.43E-16**	0.86 (0.66-1.07)	2.37
		Bindea-NK_CD56dim_cells	602	301	7.79	**6.82E-15**	0.8 (0.6-1.0)	2.22
		Bindea-NK_cells	602	301	7.51	**5.72E-14**	0.73 (0.54-0.92)	2.07
		Bindea-CD8_T_cells	602	301	7.45	**9.13E-14**	0.73 (0.54-0.92)	2.07
		Bindea-iDC	602	301	7.36	**1.86E-13**	0.7 (0.52-0.89)	2.01
		Bindea-Macrophages	602	301	6.92	**4.48E-12**	0.66 (0.48-0.85)	1.93
		Bindea-NK_CD56bright_cells	602	301	6.59	**4.44E-11**	0.62 (0.44-0.8)	1.85
		Bindea-Tcm	602	301	6.54	**6.34E-11**	0.61 (0.43-0.79)	1.83
		Bindea-DC	602	301	6.44	1.23E-10	0.59 (0.41-0.77)	1.8
		Bindea-Neutrophils	602	301	6.23	4.75E-10	0.57 (0.4-0.76)	1.77
		Bindea-T_helper_cells	602	301	6.2	5.56E-10	0.57 (0.39-0.75)	1.76
		Bindea-Th2_cells	602	301	5.51	3.63E-08	0.49 (0.32-0.66)	1.62
		Bindea-Eosinophils	602	301	4.75	2.01E-06	0.41 (0.25-0.59)	1.51
		Bindea-Mast_cells	602	301	4.58	4.72E-06	0.4 (0.23-0.57)	1.49
		Bindea-Treg	602	301	4.19	2.76E-05	0.37 (0.2-0.54)	1.44
		Bindea-pDC	602	301	4.11	4.03E-05	0.35 (0.19-0.53)	1.42
		Bindea-Th17_cells	602	301	0.51	6.13E-01	0.04 (-0.12-0.2)	1.04
		TILs-Lymphoids	602	301	11.71	**1.09E-31**	2.19 (1.84-2.58)	8.97
		TILs-TIL_score	602	301	11.52	**1.01E-30**	1.81 (1.52-2.14)	6.13
		TILs-Myeloids	602	301	9.09	**1.02E-19**	1.01 (0.8-1.24)	2.75
		TILs-Stroma	602	301	4.81	**1.50E-06**	0.44 (0.26-0.62)	1.55
		TILs-Cancer	602	301	0.15	8.82E-01	0.01 (-0.15-0.17)	1.01
		ICR	602	301	10.7	**9.80E-27**	1.62 (1.34-1.93)	5.05
		Ayers-TIS	602	301	10.64	**1.92E-26**	1.5 (1.23-1.79)	4.47
		Coppola-TLS	602	301	10.47	**1.15E-25**	1.27 (1.04-1.52)	3.57
		Rooney-Cyto_act	602	301	10.29	**7.66E-25**	1.44 (1.18-1.72)	4.22
		APMS_score	602	301	4.7	**2.56E-06**	0.41 (0.24-0.58)	1.51
		Kieffer - CAF-S1	602	301	7.07	**1.54E-12**	0.67 (0.49-0.86)	1.96
		Givel - CAF-S4	602	301	6.09	1.16E-09	0.6 (0.41-0.8)	1.82
		Biffi - iCAF	602	301	5.34	**9.11E-08**	0.48 (0.31-0.66)	1.61
		Biffi - myCAF	602	301	5.25	1.48E-07	0.46 (0.29-0.64)	1.59
CAF-S1	myCAF	Kieffer - wound-myCAF	602	301	6.05	**1.42E-09**	0.55 (0.38-0.73)	1.74
		Kieffer - ecm-myCAF	602	301	5.64	**1.71E-08**	0.51 (0.34-0.69)	1.66
		Kieffer - TGFβ-myCAF	602	301	5.54	**3.00E-08**	0.5 (0.33-0.68)	1.65
	iCAF	Kieffer - IFNγ-iCAF	602	301	9.39	**6.26E-21**	1.01 (0.81-1.23)	2.75
		Kieffer - IL-iCAF	602	301	7.8	**6.27E-15**	0.78 (0.59-0.98)	2.18

		Kieffer - detox-iCAF	602	301	6.55	**5.84E-11**	0.64 (0.45-0.83)	1.89
